# Mountain sickness in altitude inhabitants of Latin America: A systematic review and meta-analysis

**DOI:** 10.1371/journal.pone.0305651

**Published:** 2024-09-24

**Authors:** J. Pierre Zila-Velasque, Pamela Grados-Espinoza, P. Alejandra Goicochea-Romero, Gustavo Tapia-Sequeiros, J. Enrique Pascual-Aguilar, Arturo J. Ruiz-Yaringaño, Shamir Barros-Sevillano, Jhon Ayca-Mendoza, Wendy Nieto-Gutierrez

**Affiliations:** 1 Red Latinoamericana de Medicina en la Altitud e Investigación (REDLAMAI), Pasco, Peru; 2 Facultad de Ciencias de la Salud, Carrera de Medicina Humana, CHANGE Research Working Group, Universidad Científica del Sur, Lima, Peru; 3 Facultad de Ciencias de la Salud, Universidad Privada de Tacna, Tacna, Peru; 4 Sociedad Científica de San Fernando, Lima, Peru; 5 Facultad de Medicina Humana, Universidad Nacional Mayor de San Marcos, Lima, Peru; 6 Facultad de Ciencias de la Salud, Escuela de Medicina, Universidad César Vallejo, Trujillo, Perú; 7 Unidad de Investigación para la Generación de Síntesis de Evidencia en Salud, Vicerrectorado de Investigación, Universidad San Ignacio de Loyola, Lima, Peru; Universidad de Las Americas, Quito-Ecuador, ECUADOR

## Abstract

**Objective:**

Chronic and acute mountain sickness is known worldwide, but most of the available information comes from the eastern continent (Himalayas) without taking into account the west which has the most recent group located at altitude, the Andes. The aim of this study was to synthesize the evidence on the prevalence of acute and chronic mountain sickness in Latin American countries (LATAM).

**Methods:**

A systematic search of the variables of interest was performed until July 8, 2023 in the Web of Science, Scopus, PubMed and Embase databases. We included studies that assessed the prevalence of mountain sickness in high-altitude inhabitants (>1500 m.a.s.l) who lived in a place more than 12 months. These were analyzed by means of a meta-analysis of proportions. To assess sources of heterogeneity, subgroup analyses and sensitivity analyses were performed by including only studies with low risk of bias and excluding extreme values (0 or 10,000 ratio). PROSPERO (CRD42021286504).

**Results:**

Thirty-nine cross-sectional studies (10,549 participants) met the inclusion criteria. We identified 5 334 and 2 945 events out of 10,000 with acute and chronic mountain sickness in LATAM countries. The most common physiological alteration was polycythemia (2,558 events), while cerebral edema was the less common (46 events). Clinical conditions were more prevalent at high altitudes for both types of MS.

**Conclusion:**

Acute mountain sickness (AMS) occurs approximately in 5 out of 10 people at high altitude, while chronic mountain sickness (CMS) occurs in 3 out of 10. The most frequent physiological alteration was polycythemia and the least frequent was cerebral edema.

## Introduction

Latin America (LATAM) is home to the world’s most extended mountain range, which traverses Argentina, Bolivia, Chile, Colombia, Ecuador, Peru and part of Venezuela. This mountain range boasts an average altitude ranging from 1,500 to over 4,000 meters above sea level (m) [1]. Consequently, LATAM is inhabited by approximately 40 million people who reside at high altitudes, with over 5 million of them living at altitudes exceeding 4,000 m. [1, 2], making them susceptible to mountain sickness (MS).

MS is a syndrome affecting both the cerebral and pulmonary systems and occurs due to hypoxia following an initial ascent to higher altitudes [3]. The diagnosis is based on clinical evaluation, requiring the identification of characteristic hypoxic symptoms. However, these symptoms can vary in severity [4], potentially leading to conditions like pulmonary and cerebral edema, polycythemia, hemorrhage, ataxia, and even coma in some cases [5–7], and depend on the duration of the exposure [5].

Previous studies have reported the frequency of MS in different regions of the world [8–10]. However, these reports are mainly from eastern areas (such as Nepal Himalaya and Ethiopian highlands) with comparatively scarce information with other mountainous places in the world, such as the Andes [11]. In fact, a previous systematic review [12] determined a global prevalence of MS at approximately 12%; however, they omitted to include studies from LATAM despite being from regions with cities over 4000 m. with residents susceptible to MS [13–15].

Likewise, none of the reports describe the frequencies of MS according to the types of symptoms (mild and severe), exposure (acute or chronic), and complications (exaggerated pulmonary hypertension, pulmonary and cerebral edema). The absence of this information can result in the mishandling of data and a lack of appropriate solutions for managing symptoms and allocating health resources efficiently, in addition to the fact that little research from the Andean countries is evident [16]. Therefore, the present study aims to synthesize the evidence on the prevalence of acute and chronic MS in Latin American countries.

## Methods

### Protocol registration and research question

A systematic review was performed followed the guidelines of the Preferred Reporting Items for Systematic Reviews and Meta-Analyses 2020 (PRISMA) (S1 Checklist) [17] and the Cochrane Handbook [18]. The protocol of the study was registered in the International prospective register of systematic reviews—PROSPERO (CRD42021286504) [19].

The research question was “*What is the prevalence of Mountain Sickness in Altitude Inhabitants of Latin America*?*”*, with the following PECO (where the means of P: population E: exposure, O: outcome) structure: 1) population: altitude inhabitants (defined as a person who live in a place more than 12 months); 2) exposure: altitude (following the ranges of intermediate altitude 1500–2500 m, from where physiological changes are detectable), high altitude (2500–3500 m), very high altitude (3500–5800 m), extreme altitude (>5800 m.), and “death zone” (>8000 m.a.s.l.) [20]; and 3) outcome: prevalence of AMS and CMS, according to each disease and study.

### Systematic search

A systematic search was performed up to July 8, 2023 in the databases of Web of Science (WoS), Scopus, Medline (thought PubMed) and Embase. We adapted the search strategy for each of the databases using keywords related to “mountain sickness” and “Latin America”. Also, we include terms for each Latin America country that report a high altitude (Peru, Chile, Argentina, Bolivia, Venezuela, Ecuador, and Colombia), taking into account all the cities located at high altitudes. The search strategy of each database is available in S2 Table. Also, we evaluated the references of included studies to identify potentially eligible studies that weren’t found in the systematic search.

### Study selection and data extraction

**Fig 1:**
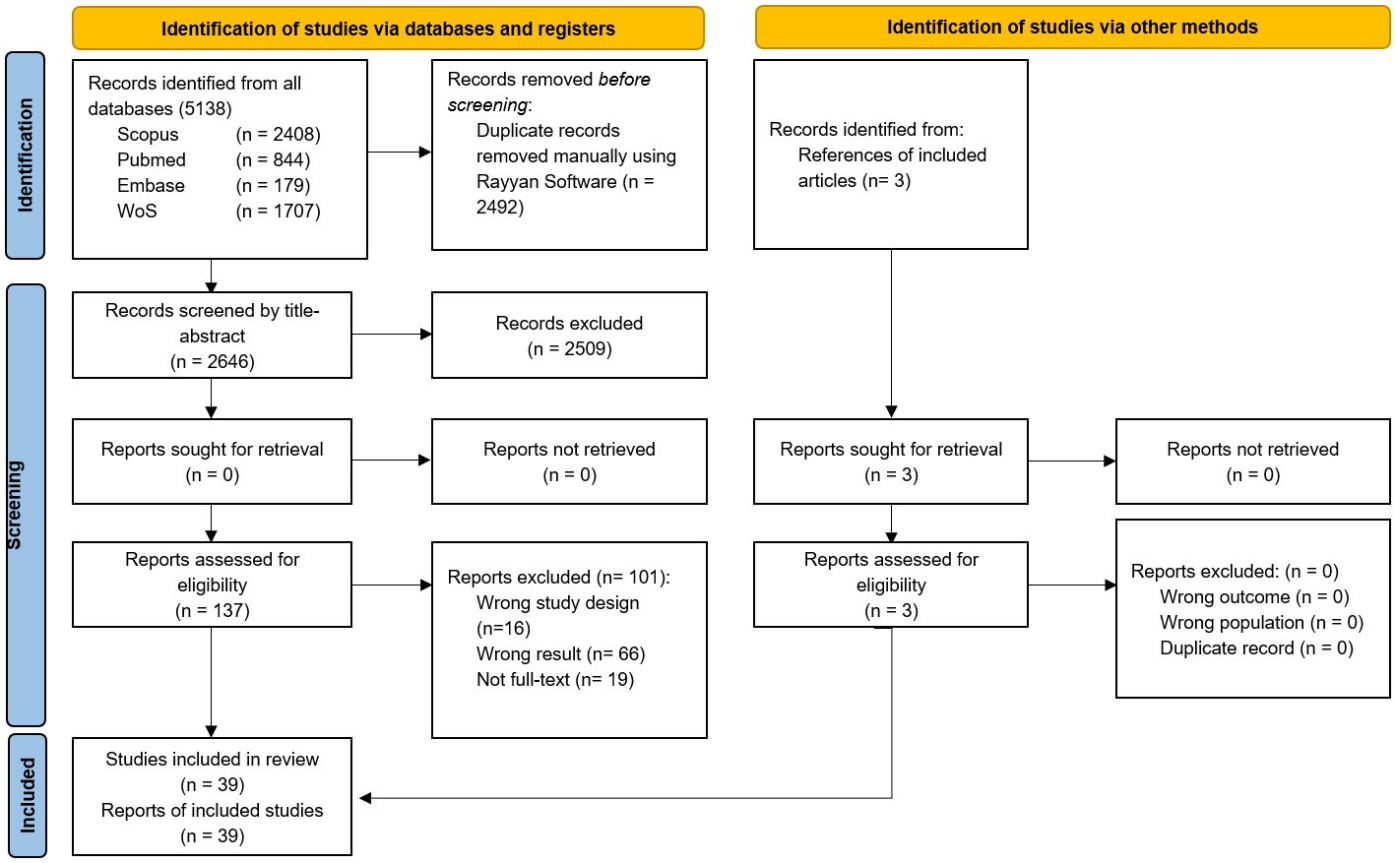
PRISMA flow chart for the study selection process.

Duplicates were removed by two independent reviewers using Endnote v.20. The study selection was performed by four blinded and independent reviewers (JEPA, AJRY, PAGR, GTS) divided into two groups of two. The selection was performed in two stages, the first, a selection to evaluate the title and abstract, and the second, to evaluated the full-text. If there were discrepancies, these were resolved by consensus. The selection process was described in Fig 1.

We included observational studies (cross-sectional or cohort studies) that report the prevalence of MS in people living at altitude in some Latin America country. We excluded editorials, commentaries, opinions, congress abstract, and reviews. No publication date or language restrictions were applied. The studies that were excluded and the reason for exclusion are described in S1 Table.

The data extraction was performed by four independent authors (JEPA, AJRY, PAGR, GTS) divided into two groups of two. We extracted variables like the first author, year of publication, study design, country, sample size, age, sex, type of resident (native: defined as a person who has been born and raised at altitude, or resident: defined as a person who leaves his or her city of birth to settle elsewhere and who at altitude has a period of stay of more than 12 months), altitude level, time of residence, place of residence (urban: defined like a place that has at least 100 houses together forming blocks or blocks and includes population centers that are district capitals and rural: defined as a place that does not meet the criteria of an urban area [21]), time of exposure, sample of people with acute and chronic mountain sickness, number of events, and type of symptoms. The discrepancies were resolved by a consensus.

### Risk of bias

Four authors (PGE, PAGR, AJRY, JAM) independently assessed the methodological quality of prevalence studies using the Joanna Briggs Institute Critical Appraisal Tool [22]. This scale has 9 items (1, Was the sample frame appropriate to address the target population? 2. Were study participants sampled appropriately?, 3. Was the sample size adequate?, 4. Have the study subjects and environment been described in detail?, 5. Has the data analysis been performed with sufficient coverage of the identified sample?, 6. Were valid methods used to identify the condition?, 7. Was the condition measured in a standard and reliable way for all participants?, 8. Was there adequate statistical analysis?, 9. Was the response rate adequate and, if not, was the low response rate adequately managed?), with possible answers of "Yes", "No" and "Unclear". The quality score was considered as one point for "Yes" and zero points for "No" and "Unclear". We estimated the overall risk of bias by considering the lowest risk of bias reported among the items (S1 Fig).

### Synthesis of the evidence

We used summary statistics to describe the studies, subjects, and outcomes. For the meta-analysis, we pooled the proportions (events over the total population) using a generalized linear mixed random model. To stabilize the variances, we applied the Freeman-Tukey Double Arcsine transformation, and we estimated the confidence intervals for individual study results using the Clopper-Pearson method. We assessed the presence of heterogeneity between studies through visual inspection of forest plots for all outcomes and supplemented this with an evaluation of the I^2^ parameter. The proportions were expressed per 10,000 population. The bias of publication was evaluated using the graphic of funnel plot.

As a priori subgroup analysis, we explored the proportion of MS across different altitudes, countries, types of symptoms (mild or severe), and complications (pulmonary edema, cerebral edema, exaggerated pulmonary hypertension, and exaggerated polycythemia). These measured outcomes were extracted as reported in the included studies, which were likely defined according to established international criteria. Sensitivity analysis was performed by including only studies with a low risk of bias and excluding extreme values (0 or 10,000 proportion). All analyses were conducted using R Studio.

## Results

### Characteristic of the studies

After eliminating duplicates, we identified 2,492 articles, of which 139 were evaluated in full text, and only 39 studies were included. The studies included evaluated a sample population of 10 549 with an altitude ranging from 2 640 in Colombia [23], to 6 487, in Argentina [24]. We did not identified studies from Brazil, Costa Rica, Cuba, Guatemala, Haiti, Honduras, Dominican Republic and Panama. Of the 39 studies, acute mountain sickness (AMS) was evaluated in 15 studies [24–38] (n = 2 945), chronic mountain sickness (CMS) in 22 [13, 23, 39–58] (n = 7 448), and both in 2 [59, 60] (n = 156). Regarding the studies that evaluated AMS, most of the studies were from Chile (n = 8 studies) and Peru/Argentina (n = 4 studies), which are the countries that contributed the most research on the subject. There was an altitude ranging from 2 700 m. [31] to 6 487 m [24]. On the other hand, most of the studies that analyzed CMS were from Peru (n = 18 studies), with an altitude ranging from 2 240 m. [51] to 5 300 m. [13] (Table 1).

**Table 1:** Characteristics of studies assessing the prevalence of acute and chronic mountain sickness in Latin America.

Author-Year	Study design	Country	Altitude level (m)	Type to exposure	Sample size (n)	Age (yr)	Female (%)	Native (%)	Urban (%)
Appenzeller O, et al. 2004 [41]	Prospective cohort	Peru	4338	Chronic	31	42.2 ± 1.5*	-	100	-
Appenzeller O, et al. 2006 [40]	Prospective cohort	Peru	4338	Chronic	9	36.9 ± 2.8*	0	100	-
Bilo G, et al. 2020 [42]	Cross-sectional	Peru	4340	Chronic	289	38.3 ± 13.2*	49.5	100	-
Brito J, et al. 2007 [59]	Cross-sectional	Chile	3550	Both		48.7 ± 2*	0	50	-
Brito J, et al. 2018 [29]	Cross-sectional	Chile	4400–4800	Acute		41.8 ± 0.7*	0	100	-
Cabello G, et al. 2017 [30]	Prospective cohort	Chile	3500	Acute		NR	12	-	-
Caravedo MA, et al. 2022 [38]	Prospective cohort	Peru	3350	Acute		21 (20–25) a	57.0	0	100
De Ferrari A, et al. 2014 [43]	Cross-sectional	Peru	3825	Chronic	1065	55.3 ± 12.6*	51	0	49
Garófilo A, et al. 2010 [31]	Prospective cohort	Argentina	2700–4300	Acute		34 (18–50) **	1.59	-	-
Gazal S, et al. 2019 [44]	Prospective cohort	Peru	4380	Chronic	312	46.8 ± 13.4*	0	100	-
Gonzales GF, et al.1998 [32]	Prospective cohort	Peru	3400	Acute		21–30 b	0	-	-
Gonzales GF, et al. 2009 [46]	Prospective cohort	Peru	4340	Chronic	41	47.3 ± 1.2*	0	100	-
Gonzales, GF, et al. 2011 [47]	Cross-sectional	Peru	4340	Chronic	41	44.7 ± 9.3*	0	100	-
Gonzales GF, et al. 2011 [58]	Cross-sectional	Peru	4340	Chronic	103	45.4 ± 0.8*	0	100	-
Gonzales GF, et al. 2013 [45]	Cross-sectional	Peru	4100	Chronic	506	35–75 b	69	-	-
Hancco I, et al. 2020 [13]	Cross-sectional	Peru	5100–5300	Chronic	1594	32 (23–39) a	14.7	0	-
Irarrazaval S, et al. 2017 [33]	Prospective cohort	Chile	3920	Acute		30.1 (24.9–32.7) a	0	-	-
Jefferson JA, et al. 2004 [60]	Cross-sectional	Peru	4300	Both		36.6 (23–58) **	-	0	100
Lang M, et al. 2021 [34]	Prospective cohort	Chile	3300	Acute		12.5 ± 1.1*	57.1	-	100
Leon-Velarde F, et al. 1993 [52]	Cross-sectional	Peru	4300	Chronic	2875	20–69 b	-	0	-
Leon-Velarde F, et al. 1994 [53]	Cross-sectional	Peru	4300	Chronic	97	NR	0	87	-
Leon-Velarde F, et al. 1997 [55]	Cross-sectional	Peru	4300	Chronic	112	37 ± 5.23*	100	0	100
Leon-Velarde F, et al. 2001 [54]	Cross-sectional	Peru	4300	Chronic	33	NR	100	0	100
Maignan M, et al. 2009 [48]	Cross-sectional	Peru	4300	Chronic	57	NR	0	0	100
Moraga FA, et al. 2002 [36]	Prospective cohort	Chile	3500–4400	Acute		NR	-	-	-
Moraga FA, et al. 2008 [35]	Prospective cohort	Chile	3500	Acute		4.3 ± 1*	43.8	-	-
Peñaloza D, et al. 1963 [61]	Cross-sectional	Peru	4540	Chronic	38	22 ± 3.8*	0	100	0
Pesce C, et al. 2005 [37]	Retrospective cohort	Argentina	5088	Acute		36.5 ± 10.1*	15.4	-	-
Quispe-Trujillo MM, et al. 2020 [50]	Cross-sectional	Peru	5200	Chronic	51	44 ± 7*	0	0	-
Riaño López L, et al. 2021 [23]	Case series	Colombia	2640	Chronic	6	11 ± 3*	16.7	0	100
Richalet JP, et al. 2002 [39]	Prospective cohort	Chile	3800–4600	Chronic	29	25 ± 5*	0	-	-
Salazar H, et al. 2012 [25]	Cross-sectional	Peru	3400	Acute		32 (25–49) a	55.2	-	-
Seoane L, et al. 2011 [26]	Prospective cohort	Argentina	3500–5000	Acute		35 (26–44) **	50.4	87.5	-
Serrano-Dueñas M. 2005 [27]	Prospective cohort	Ecuador	4100–5800	Acute		26.1 (18–41) **	35.7	0	-
Siques P, et al. 2009 [28]	Prospective cohort	Chile	3550	Acute		17.9 ± 0.1*	0	-	-
Steele AR, et al. 2021 [57]	Cross-sectional	Peru	4380	Chronic	24	40 ± 12*	0	0	100
Valencia-Flores M, et al. 2004 [51]	Prospective cohort	Mexico	2240	Chronic	57	42.7 ± 12.1*	59.6	-	100
Van Roo JD, et al. 2011 [24]	Prospective cohort	Argentina	4365–6487	Acute		42.1 (39.2–45.1) **	9.1	-	-
Vizcarra-Escobar D, et al. 2015 [56]	Cross-sectional	Peru	4100–4300	Chronic	78	NR	0	-	0

NR: No Reported, yr (year), d (day).

* Mean age ± SD.

** Mean age (range)

^a^ Median age (IQR)

^b^ Age range

Native: defined as a person who has been born and raised at altitude.

Urban: defined like a place that has at least 100 houses together forming blocks or blocks and includes population centers that are district capitals.

### Prevalence of mountain sickness

**Fig 2:**
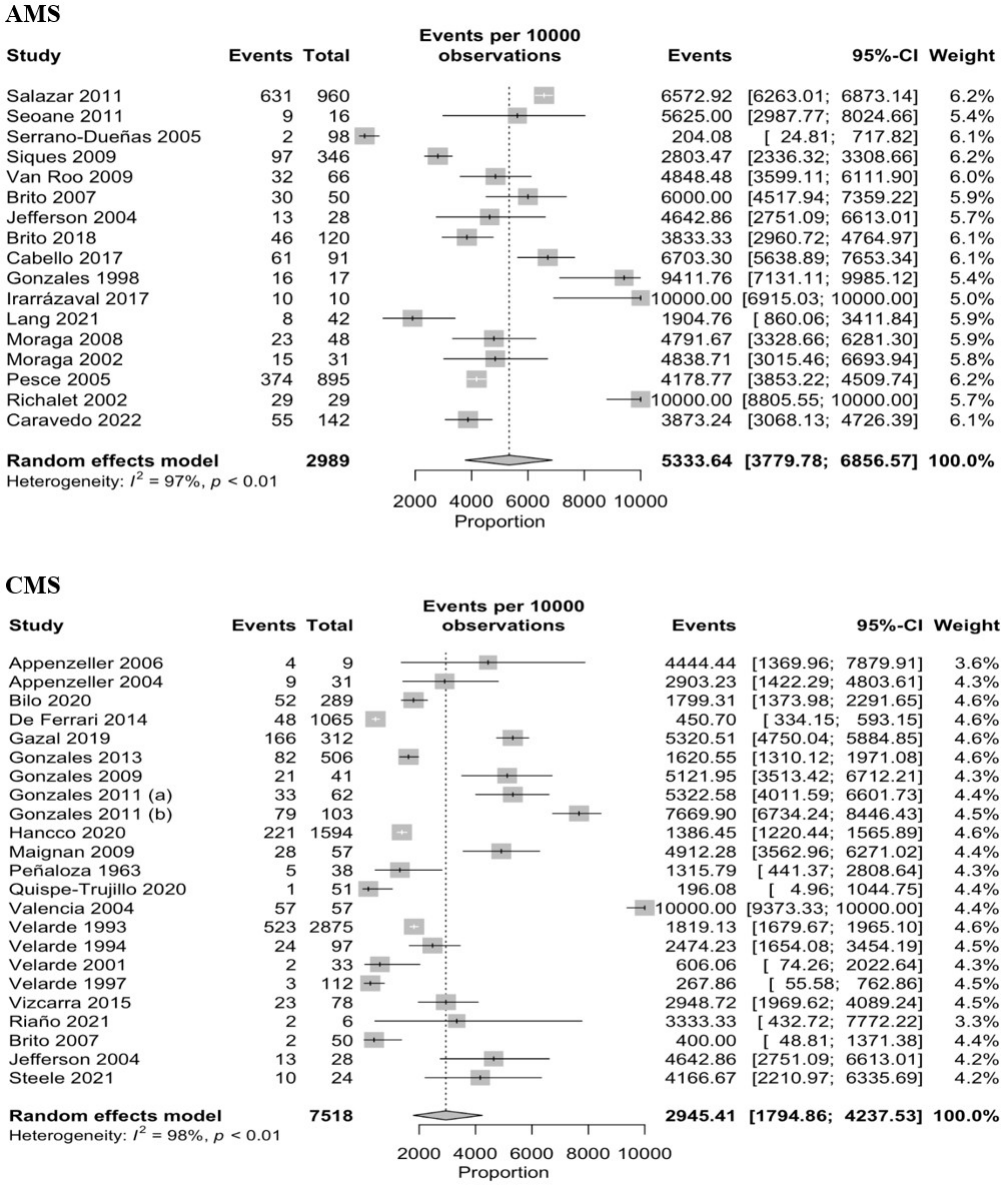
Meta-analysis on the prevalence of Acute (A) and Chronic (B) Mountain Sickness.

We identified 39 studies which it has the necessary information for analyzed the overall prevalence. We identified that in 10 000 people, 5 334 reported AMS (95% CI: 3 780–6 857, I2 = 97.0%, n = 2 989) and 2 945 reported CMS (95% CI: 1 795–4 237, I2 = 98.0%, n = 7 518) (Fig 2).

**Fig 3:**
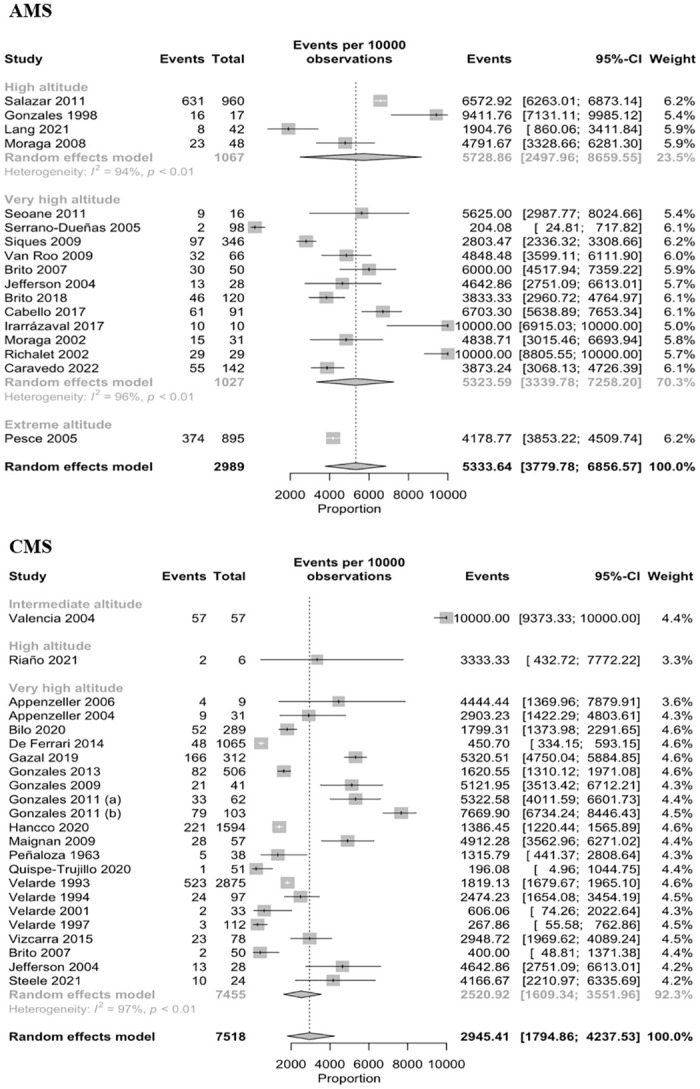
Prevalence of Acute (A) and Chronic (B) Mountain Sickness by altitude.

The number of events of AMS by 10 000 people according to the levels of altitude the events were 5 728 (95% CI: 2 497–8 659, I2 = 94.0%, n = 1 067–4 studies) at high altitude, 5 323 (95% CI: 3 339–7 258, I2 = 96.0%, n = 1 027–12 studies) at very high altitude, and 4 179 (95% CI: 3 853–4 509, n = 895–1 study) at extreme altitude. In relation to CMS, we identified that the totally of people had symptoms at intermediate altitude (n = 57) and high altitude (n = 6). However, 2 520 (95% CI: 1 609–3 551, I2 = 97.0%, n = 7 455–21 studies) events of CMS were reported in high altitude in a population of 10 000 persons (Fig 3).

**Fig 4:**
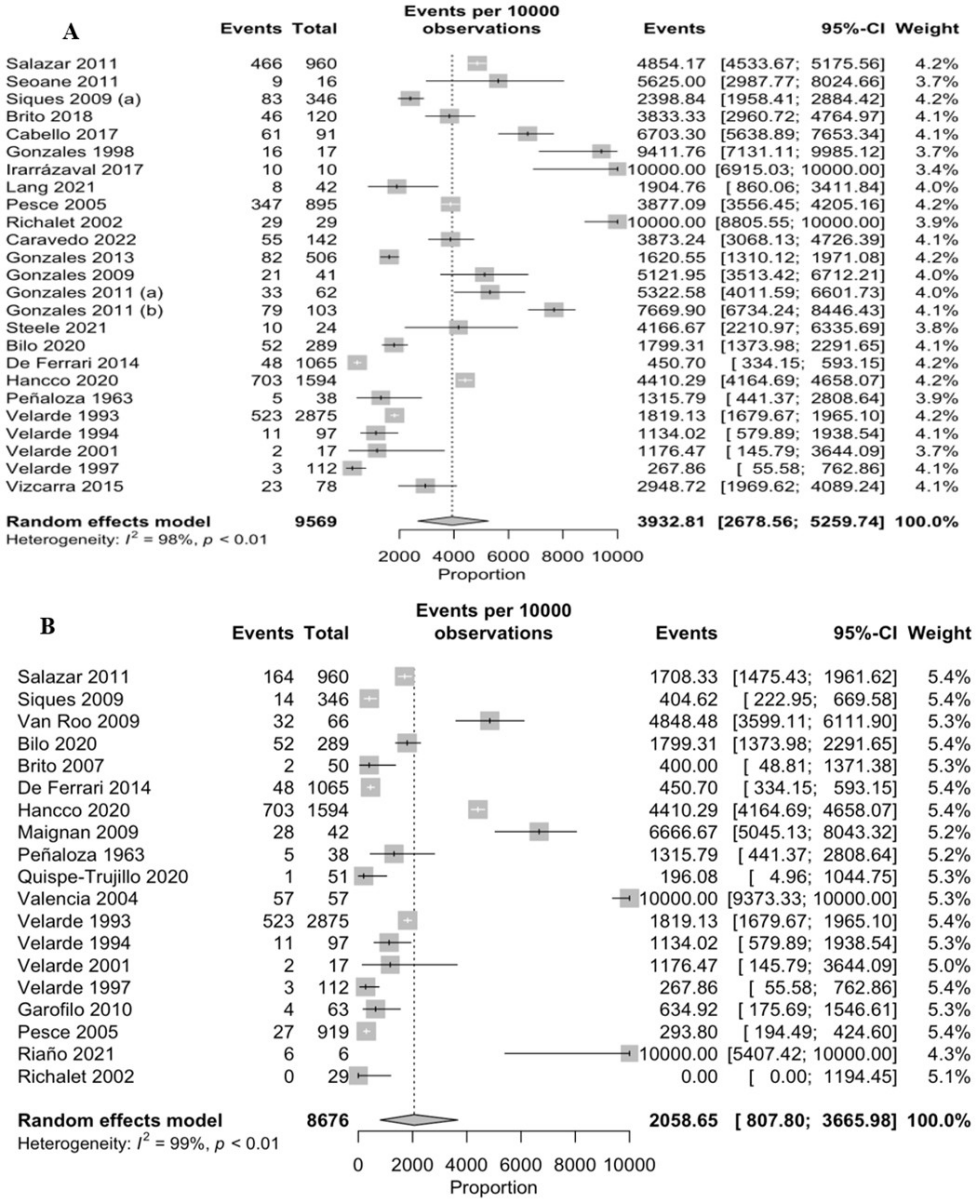
Meta-analysis on the prevalence of mild (A) and severe (B) symptoms of Mountain Sickness.

**Fig 5:**
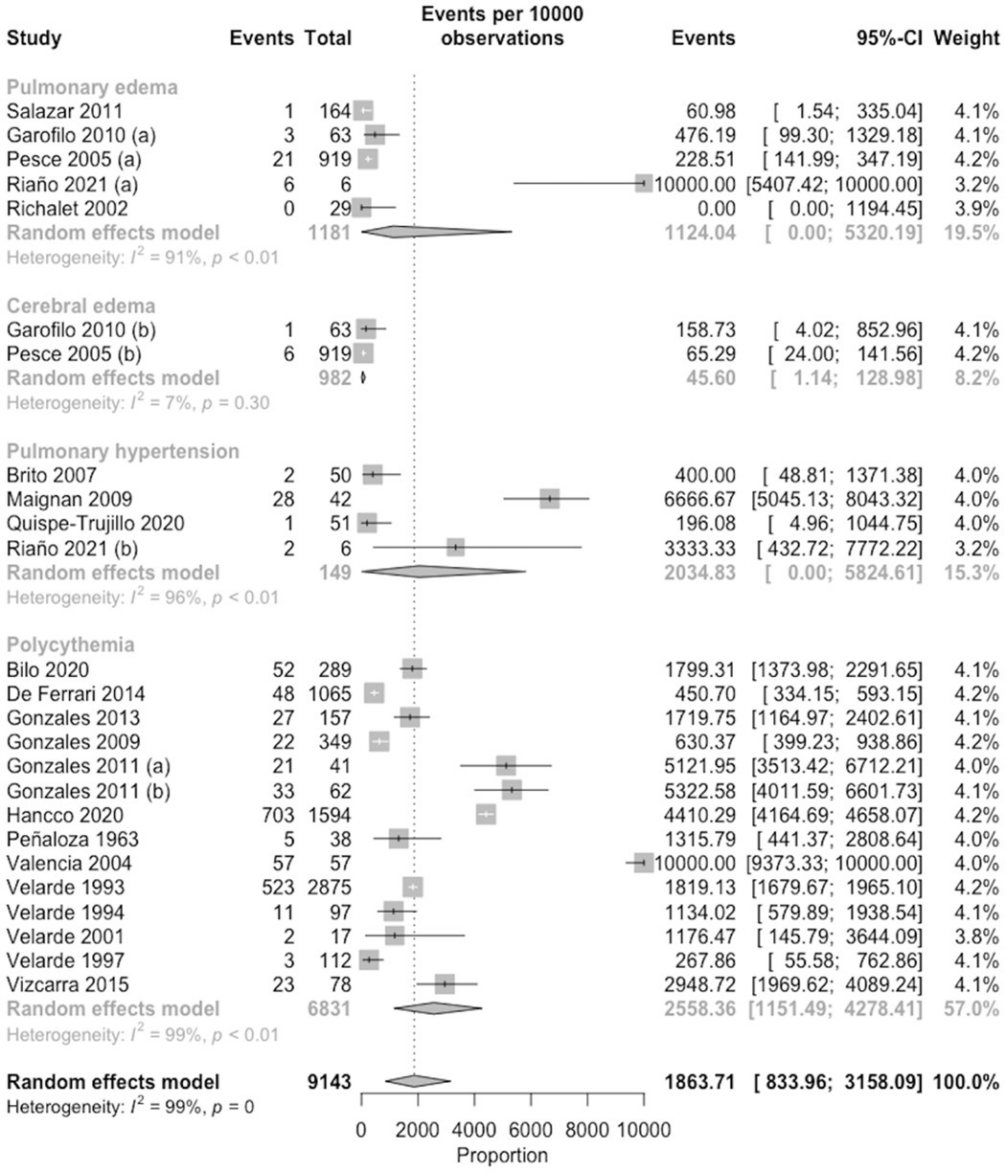
Meta-analysis on the prevalence of complications of mountain sickness.

We included 25 and 21 studies to mild (headache) and severe (pulmonary and cerebral edema, pulmonary hypertension and polycythemia) symptoms, which were reported 3 932 (95% CI: 2 678–5 259, I2 = 98.0%, n = 9 569) and 1 776 (95% CI: 675–3 220, I2 = 99.0%, n = 9 658) that in 10 000 persons, respectively (Fig 4). According to the order of frequency, we found that polycythemia was most frequent with 2 558 events (95% CI: 1 151–4 278, I2 = 99.0%, n = 6 831–14 studies), followed by pulmonary hypertension with 2 035 (95% CI: 0. 00–5 825, I2 = 96.0%, n = 149–4 studies), pulmonary edema with 1 124 (95% CI: 0.00–5 320, I2 = 91.0%, n = 1 181–5 studies) and finally cerebral edema was present in 46 events (95% CI: 1.14–129, I2 = 7.0%, n = 982–2 studies) out of 10 000 persons in all the aforementioned pathologies (Fig 5).

**Fig 6:**
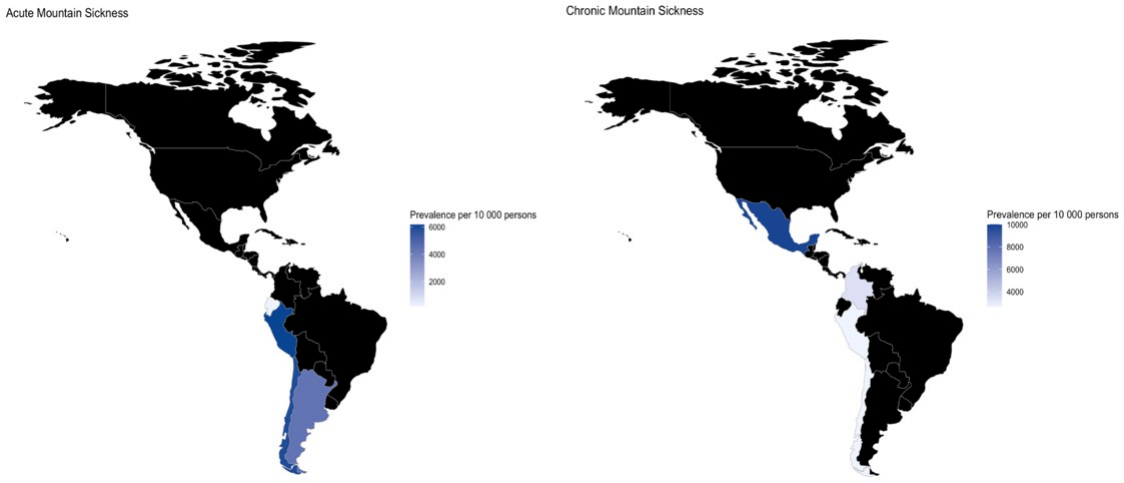
Prevalence of acute and chronic mountain sickness by country, based on the number of published studies.

In relation to the differences by country, we found that a highest prevalence of AMS was in Peru (6 171 events per 10 000 persons, CI 95% 3 628–8 425, I2 = 94%, n = 4 studies) and Chile (5 896 events per 10 000 persons, CI 95% 3 619–7 996, I2 = 95%, n = 9 studies), and for CMS was in Mexico (10 000 events per 10 000 persons, CI 95% 9 701–10 000, n = 1 study) and Colombia. (3 333 events per 10 000 persons, CI 95% 130–7 636, I2 = 95%, n = 1 study) (Fig 6).

### Risk bias assessment, publication bias, and sensitivity analysis

We assessed a high risk of bias in the overall included studies and for each outcome individually. Only three studies [28, 38, 45] are considered to have a low risk of bias in all domains, and only one [43] is classified as having an unclear risk. The domain with the highest risk was “Was the sample size adequate?” followed by “Was the response rate adequate and, if not, was the low response rate adequately managed?”. On the other hand, the domain with the lowest risk was “Have the study subjects and environment been described in detail?” (S1 Fig).

The studies with low risk of bias shows a lower prevalence of AMS (4 134 events in 10 000; 95% CI 3 836–4 437 vs 5 334 events in 10 000; 95% CI 3 780–6 857) and CMS (1 621 events in 10 000; 95% CI: 1 310–1 971 vs 2 945 events in 10 000; 95% CI: 1 795–4 237) compared with main analysis; however, their confidence intervals overlap. (S2 Fig). In the same way, the sensitivity analysis excluding the outliers shows a lower prevalence of AMS (4,526 events in 10,000; 95% CI 3,294–5,787 vs. 5,334 events in 10,000; 95% CI 3,780–6,857) and CMS (2,533 events in 10,000; 95% CI: 1,637–3,542 vs. 2,945 events in 10,000; 95% CI: 1,795–4,237) compared with the main analysis, but with overlapping confidence intervals. (S3 Fig).

We observed an asymmetry in the funnel plot for studies reporting the prevalence of acute and chronic mountain sickness, raising suspicions of publication bias (S4 Fig).

## Discussion

This systematic review focused on investigating the prevalence of MS in moderate to high altitude inhabitants in Latin America, which originates from a failure to adapt adequately to high altitudes and its severity is determined by hypoxemia according to the altitude and the time of exposure [40]. To our knowledge, this is the first study to report the prevalence of both acute and chronic MS along with each of their symptoms revealing significant variability in the prevalence of MS across different countries and altitudes in LATAM. There was a notable absence of data from certain countries in the region, suggesting the need for further research in those areas as also evidenced by a 40-year bibliometric analysis in which the south American countries with the highest altitude and the greatest number of publications were Peru, Bolivia and Colombia [16]. The results of this review show that both acute MS (AMS) and chronic MS (CMS) are significant issues in altitude populations in Latin America. The prevalence of AMS and CMS varied depending on altitude, country, and the presence of mild or severe symptoms. Overall, a higher prevalence of AMS was observed in Peru and Chile, while the prevalence of CMS was higher in Mexico and Colombia.

### Acute mountain sickness

Our study found that 5 out of 10 persons had AMS in LATAM countries, slightly higher occurrence compared to estimations for Europe (3 to 4 events out of 10 persons) [62]. However, our study involved a higher average altitude compared to the European study (3500 vs. 4,023). On the other hand, the meta-analysis included acclimatized high-altitude inhabitants of LATAM, while in Europe the majority of mountain ascents are for tourism or sport by low altitude residents [62]. AMS can manifest itself variably according to the physiological adaptation to altitude of each individual, however, a greater severity of symptoms is observed in low altitude residents who ascend to higher altitudes [63].

AMS is a complex disorder triggered by high-altitude hypoxia, initiating a pulmonary response characterized by an increase in tidal volume and respiratory rate, leading to subsequent respiratory alkalosis [63]. Simultaneously, cerebral vasodilation occurs [51], manifesting as headaches [61]. The onset of AMS typically occurs six to 12 hours after high-altitude climbing, but can manifest as quickly as within one to two hours or as late as 24 hours [64]. Given this variability, it is challenging to precisely identify the occurrences of this disease, and there is a possibility that some events may not be captured in our estimations.

The most common symptoms of AMS were headaches, while more severe complications such as pulmonary and cerebral edema, pulmonary hypertension, and polycythemia were also present, albeit to a lesser extent. These findings underscore the importance of considering a wide range of symptoms and complications when assessing the burden of MS in the region.AMS is characterized by the presence of mild symptoms [63, 65], primarily resulting from mechanisms of adaptation to hypoxemia [5, 66]. These symptoms encompass headache, fatigue, anorexia, nausea, dizziness as well as sleep disorders. Identifying the symptoms will be necessary to diagnose AMS, using the Lake Louise clinical score [64]. In our study, we identified that these symptoms were present in four of 10 people, with headaches being the most commonly reported, aligning with findings from previous reviews [67]. The occurrence of headaches in AMS can be explained by various mechanisms, including cerebral edema due to hypoxia, leading to increased intracranial pressure, the elevation of cerebral blood flow, difficulty in draining cerebral venous outflow and the activation of the trigeminal vascular system due to the release of nitrous oxide and vasodilation [5, 66]. Even a higher female biological susceptibility to MSA has been proposed, however, the few studies that have investigated it showed variability for [51, 64] and against it [63, 65]. Therefore, studies with larger population and methodological quality are recommended.

### Chronic mountain sickness

Lack of long-term altitude adaptation remains the cause of CMS. Our study identified that 3 out of 10 people had CMS, a significantly higher prevalence compared to the previous study conducted in Asia (1 event out of 10), which confirms that lack of adaptation is its genesis. However, despite the pathological mechanisms and the exact biological mechanism remains unknown [68], factors associated to the context such as environmental pollution, malnutrition, the lack in basic public health needs and poverty have also been described [66]. The diagnosis of CMS is based on the Qinghai CMS Score, which includes the evaluation of common symptoms such as shortness of breath, palpitations, sleep disturbances, heart failure, peripheral venous insufficiency, paresthesia, headache, cognitive impairment, exaggerated polycythemia and pulmonary hypertension [67].

The CMS is related with the severe symptoms, considering the mechanism involved and the chronic exposures. We identified that approximately 3 out of 10 persons has severe symptoms, higher than that reported by a study in Qinghai, China, where only 2.4% of more than 1000 residents had severe symptoms [68]. However, there are some severe symptoms such as acute pulmonary edema, that it was present in 1 event out of 10 persons, which is related to the AMS that develops approximately 4 to 12 hours after arrival [58]. The cerebral edema is also related with AMS, this has a progressive presentation and is usually associated with neurological symptoms such as decreased consciousness and/or ataxia, and can even result in coma [5, 45]. We identified lower prevalence of cerebral edema (less than 1 event out of 10 persons), but slightly lower than European estimations (1 event out of 10 persons) [46]. Other complications like exaggerated pulmonary hypertension are related to chronic exposure to altitude, being the most frequent severe symptom (2 event out of 10 persons). Our estimation differs from others reported in India where the prevalence was less than 1 event out of 10 people [33]. In relation to its origin, it is known that the chronic hypoxic stimuli of living at high altitude can cause "permanent pulmonary vascular remodeling" due to increased pulmonary vascular resistance and define a subgroup of pulmonary hypertension known as high-altitude pulmonary hypertension (HAPH) [60]. It’s important to note that complicated of this can progress to cor pulmonale and congestive heart failure [33]. Polycythemia was presented approximately 3 events out of 10 persons. Our result was different from the study conducted in Tibetans where the prevalence was 1 event out of 10 [34]. It is possible that genetic differences influence the prevalence of polycythemia, due to the variation in the levels of soluble erythropoietin receptors (sEpoR) present in the blood of each individual, which buffers the increase in hemoglobin [52]. In addition, the existence of inherited genetic factors in susceptibility to these MSA and CMS has been suggested [69, 70], and thus there may be differences in disease prevalence between native and non-native but acclimatized residents, and it would be of interest to involve them in future research.

### Implications of the results and recommendations for future studies

There are few systematic reviews related to altitude, in relation to MS in LATAM, no data on the prevalence of this pathology have been reported, however, the research found focuses on the general population without considering important variables such as the type of resident (immigrant or native), the generational period and the comorbidities that influence the development of the pathology, therefore it is suggested that future primary studies analyze variables such as quality of life, carry out studies in larger samples and at different altitudes. In the present review, we found a higher prevalence of AMS and CMS in LATAM high-altitude population compared to other high-altitude populations. However, it is worth recognizing that high-altitude regions present certain difficulties, including low coverage of essential primary health care services, unequal access to essential medicines, and an absence of programs for the prevention, treatment and management of MS. As a consequence, complications of the disease may present themselves earlier, in addition, an adaptive response to high-altitude with exaggerated polycythemia and pulmonary hypertension are associated with the development of arterial hypertension [53] and others such as the development of cognitive impairment. To limit the development of these, it is recommended to strengthen primary care through the use of protocols for the management of each pathology, improve the relationship between the community, health personnel and patients.

For future research, addressing these limitations by employing more rigorous study designs and including a greater number of studies with a low risk of bias is recommended. Furthermore, it would be beneficial to further explore risk factors and underlying mechanisms of MS in LATAM, as well as evaluate the effectiveness of prevention and treatment strategies. We suggest that future research considers including specific ethnic groups from the high-altitude regions in the Andes to facilitate genetic studies aimed at further understatement of their physiological responses to high altitude. Finally, the results presented are intended to generate adequate epidemiological information.

### Limitations and strengths of the review

Limitations in this review should be noted. First, the prevalence estimates were not standardized due to the different sample sizes of each study and the statistical methods used. Additionally, there was great heterogeneity between the studies reviewed, thus limiting their evidence certainty, and some studies did not have the necessary information to be include in the meta-analysis or to conduct subgroup analyses. Most of the studies had small sample size, which generated imprecision about individual studies that generated a low evidence certainty and a high risk of bias identified in most of the included studies, which could affect the validity and generalizability of the results. Similarly, a potential bias is the poorly defined target population in some studies, with a bias towards AMS due to variability regarding the inclusion of climbing tourists with AMS or the exclusion of long-term high-altitude residents. Finally, evidence of publication bias was observed, suggesting that studies with negative results may not have been published.

The study has some strengths as the first systematic review and meta-analysis on the prevalence of MS in LATAM that has the Andes as an altitudinal group, therefore the data presented can serve as background for future studies and the systematized information can be useful for researchers, public health professionals working in high altitude regions and the development of national strategies for the prevention and treatment of AMS and CMS in mid and high-altitude regions. Also, we address a clear gap in the literature when considering high-altitude residents in LATAM for presenting unique characteristics such as shorter residence time at altitude.

## Conclusion

This systematic review provides an overview of the prevalence of AMS in altitude inhabitants in LATAM which occurs in 5 out of 10 people at high altitude, while CMS occurs in 3 out of 10. The most frequent symptom type was polycythemia and the least frequent was cerebral edema. The results underscore the importance of addressing this public health issue and highlight the need for further research including more participants and data from the different Andean countries to obtain more representative results and a better understanding of its epidemiology and address its clinical and public health implications in the region.

## Supporting information

S1 Checklist PRISMA checklist. (DOCX)

S1 Table Excluded studies. (DOCX)

S2 Table Search strategy. (DOCX)

S1 Fig Risk of bias of included studies. (DOCX)

S2 Fig Sensitivity analysis on prevalence of Acute (A) and Chronic (B) Mountain Sickness between risk of bias. (DOCX)

S3 Fig Sensitivity analysis on prevalence of Acute (A) and Chronic (B) Mountain Sickness excluding outliers. (DOCX)

S4 Fig Publication bias on the prevalence of acute (A) and chronic (B) mountain sickness. (DOCX)

## References

[1] Andes: World’s Longest Mountain Range | Live Science. Accessed September 20, 2023. https://www.livescience.com/27897-andes-mountains.html

[2] Stuber T, Scherrer U. Circulatory adaptation to long-term high altitude exposure in Aymaras and Caucasians. *Prog Cardiovasc Dis*. 2010;52(6):534–539. doi: 10.1016/j.pcad.2010.02.009 20417347

[3] Murdoch D. Altitude sickness. *BMJ Clin Evid*. 2010;2010:1209. 21718562 PMC2907615

[4] Bärtsch P, Swenson ER. Clinical practice: Acute high-altitude illnesses. *N Engl J Med*. 2013;368(24):2294–2302. doi: 10.1056/NEJMcp1214870 23758234

[5] Garrido E, Botella de Maglia J, Castillo O. Mal de montaña de tipo agudo, subagudo y crónico. *Rev Clin Esp*. 2021;221(8):481–490. doi: 10.1016/j.rce.2019.12.013 32197780

[6] Hackett PH, Roach RC. High-Altitude Illness. *N Engl J Med*. 2001;345(2):107–114. doi: 10.1056/NEJM200107123450206 11450659

[7] Guijarro Morales A, Gil Extremera B, Maldonado Martín A. Acute mountain sickness. *An Med Interna*. 1990;7(7):375–378. 2103253

[8] Maggiorini M, Bühler B, Walter M, Oelz O. Prevalence of acute mountain sickness in the Swiss Alps. *BMJ*. 1990;301(6756):853–855. doi: 10.1136/bmj.301.6756.853 2282425 PMC1663993

[9] Vargas D M, Osorio F J, Jiménez E D, et al. Mal agudo de montaña a 3.500 y 4.250 m: Un estudio de la incidencia y severidad de la sintomatología. *Revista médica de Chile*. 2001;129(2):166–172. doi: 10.4067/S0034-98872001000200007 11351468

[10] Murdoch null. Altitude Illness Among Tourists Flying to 3740 Meters Elevation in the Nepal Himalayas. *J Travel Med*. 1995;2(4):255–256. doi: 10.1111/j.1708-8305.1995.tb00671.x 9815403

[11] Xing G, Qualls C, Huicho L, et al. Adaptation and Mal-Adaptation to Ambient Hypoxia; Andean, Ethiopian and Himalayan Patterns. *PLOS ONE*. 2008;3(6):e2342. doi: 10.1371/journal.pone.0002342 18523639 PMC2396283

[12] Meier D, Collet TH, Locatelli I, et al. Does This Patient Have Acute Mountain Sickness?: The Rational Clinical Examination Systematic Review. *JAMA*. 2017;318(18):1810–1819. doi: 10.1001/jama.2017.16192 29136449

[13] Hancco I, Bailly S, Baillieul S, et al. Excessive Erythrocytosis and Chronic Mountain Sickness in Dwellers of the Highest City in the World. *Front Physiol*. 2020;11:773. doi: 10.3389/fphys.2020.00773 32760289 PMC7373800

[14] Champigneulle B, Brugniaux JV, Stauffer E, et al. Expedition 5300: limits of human adaptations in the highest city in the world. *The Journal of Physiology*. 2023;n/a(n/a). doi: 10.1113/JP284550 38146929

[15] Monge C. Life in the Andes and Chronic Mountain Sickness. *Science*. 1942;95(2456):79–84. doi: 10.1126/science.95.2456.79 17757318

[16] Zila-Velasque JP, Grados-Espinoza P, Morán-Mariños C, Morales Pocco KO, Capcha-Jimenez US, Ortiz-Benique ZN. Adaptation and altitude sickness: A 40-year bibliometric analysis and collaborative networks. *Frontiers in Public Health*. 2023;11. Accessed November 2, 2023. https://www.frontiersin.org/articles/10.3389/fpubh.2023.1069212 36935697 10.3389/fpubh.2023.1069212 PMC10018125

[17] Page MJ, McKenzie JE, Bossuyt PM, et al. The PRISMA 2020 statement: an updated guideline for reporting systematic reviews. *BMJ*. 2021;372:n71. doi: 10.1136/bmj.n71 33782057 PMC8005924

[18] Cochrane Handbook for Systematic Reviews of Interventions. Accessed October 9, 2022. https://training.cochrane.org/handbook

[19] Zila-Velasque JP, Grados-Espinoza P, Goicochea-Romero PA, et al. Prevalence of acute and chronic mountain sickness in Latin America: A systematic review and meta-analysis. 2021. Accessed October 9, 2022. https://www.crd.york.ac.uk/prospero/display_record.php?RecordID=286504 10.1371/journal.pone.0305651 PMC11421813 39316567

[20] Imray C, Booth A, Wright A, Bradwell A. Acute altitude illnesses. *BMJ*. 2011;343:d4943. doi: 10.1136/bmj.d4943 21844157

[21] INEI. INEI—Variables Contextuales. Accessed June 20, 2023. https://www.inei.gob.pe/media/MenuRecursivo/publicaciones_digitales/Est/Lib0014/varicont.htm

[22] Munn Z, Moola S, Lisy K, Riitano D, Tufanaru C. Methodological guidance for systematic reviews of observational epidemiological studies reporting prevalence and cumulative incidence data. *Int J Evid Based Healthc*. 2015;13(3):147–153. doi: 10.1097/XEB.0000000000000054 26317388

[23] Riaño López L, Figueredo R, Vásquez-Hoyos P. Reentry High-Altitude Pulmonary Edema in Pediatric Patients. *Andes Pediatr*. 2021;92(2):257–262. doi: 10.32641/andespediatr.v92i2.2977 34106165

[24] Roo JDV, Lazio MP, Pesce C, Malik S, Courtney DM. Visual Analog Scale (VAS) for Assessment of Acute Mountain Sickness (AMS) on Aconcagua. *Wilderness & Environmental Medicine*. 2011;22(1):7–14. doi: 10.1016/j.wem.2010.10.002 21377113

[25] Salazar H, Swanson J, Mozo K, White AC, Cabada MM. Acute mountain sickness impact among travelers to Cusco, Peru. *J Travel Med*. 2012;19(4):220–225. doi: 10.1111/j.1708-8305.2012.00606.x 22776382

[26] Seoane L, Nervi R, Seoane M, Rico F, Torres S, Rodriguez M. Acute mountain sickness: Predictors of climbers’ performance at high altitudes. *Emergencias*. 2011;23:276–282.

[27] Serrano-Dueñas M. High altitude headache. A prospective study of its clinical characteristics. *Cephalalgia*. 2005;25(12):1110–1116. doi: 10.1111/j.1468-2982.2005.00968.x 16305599

[28] Siqués P, Brito J, Banegas JR, et al. Blood pressure responses in young adults first exposed to high altitude for 12 months at 3550 m. *High Alt Med Biol*. 2009;10(4):329–335. doi: 10.1089/ham.2008.1103 20039813

[29] Brito J, Siques P, López R, et al. Long-Term Intermittent Work at High Altitude: Right Heart Functional and Morphological Status and Associated Cardiometabolic Factors. *Frontiers in Physiology*. 2018;9. Accessed September 4, 2022. https://www.frontiersin.org/articles/10.3389/fphys.2018.00248 29623044 10.3389/fphys.2018.00248 PMC5874329

[30] Cabello G, Fariña E, Jeldres A, Rimoldi SF, Scherrer U. Andean High-Altituden Ancestry does not protect from acute Mountain Sickness and Altitude-Induced arterial hypoxemia. *Interciencia*. 2017;42(1):39–43.

[31] Garófoli A, Montoya P, Elías C, Benzo R. Ejercicio y la detección del Mal de Montaña Agudo de Montaña Grave. *Medicina (B Aires)*. 2010;70(1):3–7. 20228017 PMC3402082

[32] Gonzales GF, Villena A, Aparicio R. Acute mountain sickness: Is there a lag period before symptoms? *Am J Hum Biol*. 1998;10(5):669–677. doi: 10.1002/(SICI)1520-6300(1998)10:5&lt;669::AID-AJHB13&gt;3.0.CO;2-6 28561547

[33] Irarrázaval S, Allard C, Campodónico J, et al. Oxidative Stress in Acute Hypobaric Hypoxia. *High Alt Med Biol*. 2017;18(2):128–134. doi: 10.1089/ham.2016.0119 28326844

[34] Lang M, Vizcaíno-Muñoz G, Jopia P, Silva-Urra J, Viscor G. Physiological Responses at Rest and Exercise to High Altitude in Lowland Children and Adolescents. *Life*. 2021;11(10). doi: 10.3390/life11101009 34685380 PMC8541065

[35] Moraga FA, Pedreros CP, Rodríguez CE. Acute mountain sickness in children and their parents after rapid ascent to 3500 m (Putre, Chile). *Wilderness Environ Med*. 2008;19(4):287–292. doi: 10.1580/06-WEME-BR-084.1 19099320

[36] Moraga FA, Osorio JD, Vargas ME. Acute Mountain Sickness in Tourists with Children at Lake Chungará (4400 m) in Northern Chile. *Wilderness & Environmental Medicine*. 2002;13(1):31–35. doi: 10.1580/1080-6032(2002)013[0031:AMSITW]2.0.CO;2 11929059

[37] Pesce C, Leal C, Pinto H, et al. Determinants of acute mountain sickness and success on Mount Aconcagua (6962 m). *High Alt Med Biol*. 2005;6(2):158–166. doi: 10.1089/ham.2005.6.158 16060850

[38] Caravedo MA, Mozo K, Morales ML, et al. Risk factors for acute mountain sickness in travellers to Cusco, Peru: coca leaves, obesity and sex. *Journal of Travel Medicine*. 2022;29(5):taab102. doi: 10.1093/jtm/taab102 34230961

[39] Richalet JP, Donoso MV, Jiménez D, et al. Chilean miners commuting from sea level to 4500 m: a prospective study. *High Alt Med Biol*. 2002;3(2):159–166. doi: 10.1089/15270290260131894 12162860

[40] Appenzeller O, Claydon VE, Gulli G, et al. Cerebral Vasodilatation to Exogenous NO Is a Measure of Fitness for Life at Altitude. *Stroke*. 2006;37(7):1754–1758. doi: 10.1161/01.STR.0000226973.97858.0b 16763189

[41] Appenzeller O, Passino C, Roach R, et al. Cerebral vasoreactivity in Andeans and headache at sea level. *J Neurol Sci*. 2004;219(1–2):101–106. doi: 10.1016/j.jns.2003.12.014 15050445

[42] Bilo G, Acone L, Anza-Ramírez C, et al. Office and Ambulatory Arterial Hypertension in Highlanders: HIGHCARE-ANDES Highlanders Study. *Hypertension*. 2020;76(6):1962–1970. doi: 10.1161/HYPERTENSIONAHA.120.16010 33175629

[43] De Ferrari A, Miranda JJ, Gilman RH, et al. Prevalence, Clinical Profile, Iron Status, and Subject-Specific Traits for Excessive Erythrocytosis in Andean Adults Living Permanently at 3,825 Meters Above Sea Level. *Chest*. 2014;146(5):1327–1336. doi: 10.1378/chest.14-0298 24874587 PMC4219344

[44] Gazal S, Espinoza JR, Austerlitz F, et al. The Genetic Architecture of Chronic Mountain Sickness in Peru. *Frontiers in Genetics*. 2019;10. Accessed September 4, 2022. https://www.frontiersin.org/articles/10.3389/fgene.2019.00690 10.3389/fgene.2019.00690 PMC6682665 31417607

[45] Gonzales GF, Rubio J, Gasco M. Chronic mountain sickness score was related with health status score but not with hemoglobin levels at high altitudes. *Respir Physiol Neurobiol*. 2013;188(2):152–160. doi: 10.1016/j.resp.2013.06.006 23770310 PMC3752419

[46] Gonzales GF, Gasco M, Tapia V, Gonzales-Castañeda C. High serum testosterone levels are associated with excessive erythrocytosis of chronic mountain sickness in men. *Am J Physiol Endocrinol Metab*. 2009;296(6):E1319–E1325. doi: 10.1152/ajpendo.90940.2008 19318512 PMC2692401

[47] Gonzales GF, Tapia V, Gasco M, Gonzales-Castañeda C. Serum testosterone levels and score of chronic mountain sickness in Peruvian men natives at 4340 m. *Andrologia*. 2011;43(3):189–195. doi: 10.1111/j.1439-0272.2010.01046.x 21486396

[48] Maignan M, Rivera-Ch M, Privat C, Leòn-Velarde F, Richalet JP, Pham I. Pulmonary pressure and cardiac function in chronic mountain sickness patients. *Chest*. 2009;135(2):499–504. doi: 10.1378/chest.08-1094 18719057

[49] Peñaloza D, Sime F, Banchero N, Gamboa R, Cruz J, Marticorena E. Pulmonary hypertension in healthy men born and living at high altitudes. *The American Journal of Cardiology*. 1963;11(2):150–157. doi: 10.1016/0002-9149(63)90055-9 13992990

[50] Quispe-Trujillo MM, Tejada-Flores J, Ochoa-Torres D, et al. Alteraciones cardiovasculares en pacientes con eritrocitosis excesiva en residentes a 5 200 metros sobre el nivel del mar. *Acta Med Peru*. 2020;37(4). doi: 10.35663/amp.2020.374.1042

[51] Valencia-Flores M, Rebollar V, Santiago V, et al. Prevalence of pulmonary hypertension and its association with respiratory disturbances in obese patients living at moderately high altitude. *Int J Obes Relat Metab Disord*. 2004;28(9):1174–1180. doi: 10.1038/sj.ijo.0802726 15224125

[52] León-Velarde F, Arregui A, Monge C, Ruiz y Ruiz H. Aging at high altitudes and the risk of chronic mountain sickness. *Journal of Wilderness Medicine*. 1993;4(2):183–188. doi: 10.1580/0953-9859-4.2.183

[53] León-Velarde F, Arregui A, Vargas M, Huicho L, Acosta R. Chronic Mountain Sickness and Chronic Lower Respiratory Tract Disorders. *CHEST*. 1994;106(1):151–155. doi: 10.1378/chest.106.1.151 8020264

[54] León-Velarde F, Rivera-Chira M, Tapia R, Huicho L, Monge-C C. Relationship of ovarian hormones to hypoxemia in women residents of 4,300 m. *American Journal of Physiology-Regulatory*, *Integrative and Comparative Physiology*. 2001;280(2):R488–R493. doi: 10.1152/ajpregu.2001.280.2.R488 11208579

[55] León-Velarde F, Ramos MA, Hernández JA, et al. The role of menopause in the development of chronic mountain sickness. *Am J Physiol*. 1997;272(1 Pt 2):R90–94. doi: 10.1152/ajpregu.1997.272.1.R90 9038995

[56] Vizcarra-Escobar D, Mendiola-Yamasato A, Risco-Rocca J, et al. Is restless legs syndrome associated with chronic mountain sickness? *Sleep Med*. 2015;16(8):976–980. doi: 10.1016/j.sleep.2015.03.013 26026624

[57] Steele AR, Tymko MM, Meah VL, et al. Global REACH 2018: volume regulation in high-altitude Andeans with and without chronic mountain sickness. *Am J Physiol Regul Integr Comp Physiol*. 2021;321(3):R504–R512. doi: 10.1152/ajpregu.00102.2021 34346722

[58] Gonzales GF, Tapia V, Gasco M, Rubio J, Gonzales-Castañeda C. High serum zinc and serum testosterone levels were associated with excessive erythrocytosis in men at high altitudes. *Endocrine*. 2011;40(3):472–480. doi: 10.1007/s12020-011-9482-1 21553128

[59] Brito J, Siqués P, León-Velarde F, De La Cruz JJ, López V, Herruzo R. Chronic intermittent hypoxia at high altitude exposure for over 12 years: assessment of hematological, cardiovascular, and renal effects. *High Alt Med Biol*. 2007;8(3):236–244. doi: 10.1089/ham.2007.8310 17824824

[60] Jefferson JA, Simoni J, Escudero E, et al. Increased oxidative stress following acute and chronic high altitude exposure. *High Alt Med Biol*. 2004;5(1):61–69. doi: 10.1089/152702904322963690 15072717

[61] Penaloza D, Arias-Stella J. The heart and pulmonary circulation at high altitudes: healthy highlanders and chronic mountain sickness. *Circulation*. 2007;115(9):1132–1146. doi: 10.1161/CIRCULATIONAHA.106.624544 17339571

[62] Berger MM, Hüsing A, Niessen N, et al. Prevalence and knowledge about acute mountain sickness in the Western Alps. *PLOS ONE*. 2023;18(9):e0291060. doi: 10.1371/journal.pone.0291060 37708123 PMC10501682

[63] Mairer K, Wille M, Burtscher M. The prevalence of and risk factors for acute mountain sickness in the Eastern and Western Alps. *High Alt Med Biol*. 2010;11(4):343–348. doi: 10.1089/ham.2010.1039 21190503

[64] Croughs M, Van Gompel A, Van den Ende J. Acute mountain sickness in travelers who consulted a pre-travel clinic. *J Travel Med*. 2011;18(5):337–343. doi: 10.1111/j.1708-8305.2011.00537.x 21896098

[65] Wang SH, Chen YC, Kao WF, et al. Epidemiology of acute mountain sickness on Jade Mountain, Taiwan: an annual prospective observational study. *High Alt Med Biol*. 2010;11(1):43–49. doi: 10.1089/ham.2009.1063 20367488

[66] Rivera-Ch M, Castillo A, Huicho L. Hypoxia and other environmental factors at high altitude. *International Journal of Environment and Health*. 2008;2(1):92–106. doi: 10.1504/IJEnvH.2008.018675

[67] León-Velarde F, Maggiorini M, Reeves JT, et al. Consensus statement on chronic and subacute high altitude diseases. *High Alt Med Biol*. 2005;6(2):147–157. doi: 10.1089/ham.2005.6.147 16060849

[68] Jiang C, Chen J, Liu F, et al. Chronic mountain sickness in Chinese Han males who migrated to the Qinghai-Tibetan plateau: application and evaluation of diagnostic criteria for chronic mountain sickness. *BMC Public Health*. 2014;14(1):701. doi: 10.1186/1471-2458-14-701 25007716 PMC4227059

[69] Liu Z, Chen H, Xu T, Wang X, Yao C. HSPA1A gene polymorphism rs1008438 is associated with susceptibility to acute mountain sickness in Han Chinese individuals. *Mol Genet Genomic Med*. 2020;8(8):e1322. doi: 10.1002/mgg3.1322 32478477 PMC7434611

[70] MacInnis MJ, Koehle MS. Evidence for and Against Genetic Predispositions to Acute and Chronic Altitude Illnesses. *High Alt Med Biol*. 2016;17(4):281–293. doi: 10.1089/ham.2016.0024 27500591

