# Supplementary material for: Mountain sickness in altitude inhabitants of Latin America: A systematic review and meta-analysis
Source: PLoS One. 2024 Sep 24;19(9):e0305651. doi: 10.1371/journal.pone.0305651 (PMC11421813; doi:10.1371/journal.pone.0305651)
Supplement: S1 Table — (DOCX) [file pone.0305651.s002.docx]

## S1 Table. Excluded studies

| **Author** | **Tittle** | **Reason** |
| --- | --- | --- |
| Appenzeller 2002 | Acral paresthesias in the Andes and neurology at sea level | Wrong outcome |
|  | Activated coagulation at high altitude | Wrong outcome |
| Ju 2021 | Acute Altitude Acclimatization in Young Healthy Volunteers: Nocturnal Oxygenation Increases over Time, Whereas Periodic Breathing Persists | Wrong outcome |
| Saldias 1995 | Acute altitude sickness and ventilatory function in subjects intermittently exposed to hypobaric hypoxia | Wrong outcome |
| Serrano-Duenas 2000 | Acute mountain sickness: Clinical characteristics of a cohort of 615 patients | Wrong outcome |
| Zubieta-Calleja 2007 | Altitude adaptation through hematocrit changes | Wrong outcome |
| Claydon 2004 | Cardiovascular responses to orthostatic stress in healthy altitude dwellers, and altitude residents with chronic mountain sickness | Wrong outcome |
| Caparo 2013 | Cerebral blood flow velocity and autoregulation in adolescents at three different altitudes | Wrong outcome |
| Pichler-Hefti 2010 | Changes of coagulation parameters during high altitude expedition | Wrong outcome |
| Appenzeller 2006 | Chronic hypoxia in Andeans; are there lessons for neurology at sea level? | Wrong outcome |
| Socola 2010 | Chronic mountain sickness is related with exposition to biomass fuel combustion | Wrong outcome |
| Aeberli 2013 | Disturbed eating at high altitude: Influence of food preferences, acute mountain sickness and satiation hormones | Wrong outcome |
| Fruehauf 2019 | Evaluation of Acute Mountain Sickness by Unsedated Transnasal Esophagogastroduodenoscopy at High Altitude | Wrong outcome |
| Bailey 2019 | Exaggerated systemic oxidative-inflammatory-nitrosative stress in chronic mountain sickness is associated with cognitive decline and depression | Wrong type of study |
| Appenzeller 2003 | Gene expression in the Andes; relevance to neurology at sea level | Wrong outcome |
| Appenzeller 2006 | Gene expression, autonomic function and chronic hypoxia: Lessons from the Andes | Wrong type of study |
| Mejia 2005 | Genetic association analysis of chronic mountain sickness in an Andean high-altitude population | Not full-text |
| Gazal 2017 | Genetic risk factors of chronic mountain sickness | Wrong outcome |
| Moraga 2007 | Ginkgo biloba decreases acute mountain sickness in people ascending to high altitude at OllagÃ¼e (3696 m) in Northern Chile | Wrong outcome |
|  | Global Reach 2018 Heightened -Adrenergic Signaling Impairs Endothelial Function during Chronic Exposure to Hypobaric Hypoxia | Wrong type of study |
| Simpson 2021 | Global REACH 2018: Andean highlanders, chronic mountain sickness and the integrative regulation of resting blood pressure | Not full-text |
|  | Global REACH 2018: Dysfunctional extracellular microvesicles in Andean highlander males with excessive erythrocytosis | Wrong type of study |
| Tremblay 2019 | Global REACH 2018: High Blood Viscosity and Hemoglobin Concentration Contribute to Reduced Flow-Mediated Dilation in High-Altitude Excessive Erythrocytosis | Wrong type of study |
| DeSouza 2018 | Global REACH 2018: Influence of excessive erythrocytosis on coagulation and fibrinolytic factors in Andean highlanders | Wrong type of study |
| Coombs 2021 | Global Reach 2018: Nitric oxide-mediated cutaneous vasodilation is reduced in chronic, but not acute, hypoxia independently of enzymatic superoxide formation | Wrong type of study |
| Tremblay 2019 | Global Reach 2018: reduced flow-mediated dilation stimulated by sustained increases in shear stress in high-altitude excessive erythrocytosis | Wrong type of study |
| Steele 2020 | Global REACH 2018: Renal oxygen delivery is maintained during early acclimatization to 4,330 m | Wrong type of study |
| Hansen 2021 | Global REACH 2018: the adaptive phenotype to life with chronic mountain sickness and polycythaemia | Wrong type of study |
| Tymko 2020 | Global REACH 2018: The carotid artery diameter response to the cold pressor test is governed by arterial blood pressure during normoxic but not hypoxic conditions in healthy lowlanders and Andean highlanders | Wrong type of study |
| Steele 2021 | Global REACH 2018: volume regulation in high-altitude Andeans with and without chronic mountain sickness | Wrong type of study |
| Schutte 1983 | Growth of lowland native children of European Ancestry during sojourn at high altitude (3,200 m) | Wrong outcome |
| Munoz 2021 | Health Effects of Chronic Intermittent Hypoxia at a High Altitude among Chilean Miners: Rationale, Design, and Baseline Results of a Longitudinal Study | Not full-text |
| Amaru 2009 | Hematopoietic progenitor cells from patients with chronic mountain sickness lack the JAK2V617F mutation, show hypersensitivity to erythropoietin and are inhibited by statins | Wrong outcome |
| Gonzales 2011 | Hemoglobin and testosterone: Importance on high altitude acclimatization and adaptation | Wrong outcome |
| Mortola 1995 | Hering-Breuer reflexes in high-altitude infants | Not full-text |
|  | High altitude brain edema: Report of 12 patients | Wrong outcome |
| Cabada 2010 | High altitude itineraries | Wrong outcome |
| Serrano-Duenas 2007 | High-altitude headache | Wrong outcome |
| Vizcardo-Galindo 2020 | High-altitude hypoxia decreases plasma erythropoietin soluble receptor (SEPOR) concentration in humans | Wrong outcome |
| Vizcardo-Galindo 2020 | High-Altitude Hypoxia Decreases Plasma Erythropoietin Soluble Receptor Concentration in Lowlanders | Wrong outcome |
| Villca 2021 | High-altitude Illnesses and Air Travel: Pediatric Considerations | Wrong outcome |
| Zubieta-Calleja 2006 | Hypoventilation in Chronic Mountain Sickness: A mechanism to preserve energy | Wrong outcome |
| Loeppky 2008 | Hypoxemia and acute mountain sickness: which comes first? | Wrong outcome |
|  | Improved oxygenation relieves baroreflex dysfunction in Andean altitude natives with chronic mountain sickness | Wrong outcome |
| Pineda-Reyes 2020 | Incidence of acute mountain sickness and healthcare related behaviors among travelers to Cusco, Peru | Wrong outcome |
| Bermudez 2020 | Increased hypoxic proliferative response and gene expression in erythroid progenitor cells of Andean highlanders with chronic mountain sickness | Wrong outcome |
| Bermudez 2020 | Increased hypoxic proliferative response in pbmcs-derived erythroid progenitor cells of andean highlanders with chronic mountain sickness | Wrong outcome |
| Rexhaj 2011 | Increasing respiratory dead space improves sleep disordered breathing and hypoxemia in patients with chronic mountain sickness | Wrong outcome |
| Raj 2018 | Indigenous uses of ethnomedicinal plants among forest-dependent communities of Northern Bengal, India | Wrong outcome |
| Heinrich 2018 | Interleukin 6 Is Elevated in Andean Highlanders with Chronic Mountain Sickness | Wrong type of study |
| Talbot 2011 | Intravenous iron supplementation may protect against acute mountain sickness: A randomized, double-blinded, placebo-controlled trial | Wrong type of study |
| Dedobbeleer 2015 | Left ventricular adaptation to high altitude: speckle tracking echocardiography in lowlanders, healthy highlanders and highlanders with chronic mountain sickness | Wrong outcome |
| Monge 1942 | Life in the Andes and chronic mountain sickness | Wrong outcome |
| Hurtado 1966 | Man and altitude | Not full-text |
|  | Medical data from aconcagua provincial park's medical service, 2011-2012 season | Wrong outcome |
|  | Medical problems of Israelis travelling to tropical countries | Wrong outcome |
| Martinez 2006 | Medicinal plants used in Chile for the treatment of hypertension and mountain sickness | Wrong outcome |
| Gore 2013 | Methods of the international study on soccer at altitude 3600 m (ISA3600) | Not full-text |
| Arregui 1994 | Migraine, polycythemia and chronic mountain sickness | Wrong outcome |
| Cosio 1969 | Mining Work in High Altitude | Not full-text |
| Zhao 2018 | Mitochondrial dysfunction in iPSC-derived neurons in subjects with Chronic Mountain Sickness | Not full-text |
| Wachsmuth 2019 | Modification of the CO-rebreathing method to determine haemoglobin mass and blood volume in patients suffering from chronic mountain sickness | Not full-text |
| Martinez-Carpio 2003 | Mountain medicine in 2002 (United Nations' International Year of Mountains). Past, present and future | Wrong outcome |
| DiPasquale 2017 | Moving the Debate Forward: Are Normobaric and Hypobaric Hypoxia Interchangeable in the Study of Altitude? | Wrong outcome |
|  | N terminal-pro BNP (NT-proBNP) plasmatic levels variation in climbers to 2800-3500m ASL | Wrong type of study |
| Richardson 2011 | Neurophysiological evidence for cognitive and brain functional adaptation in adolescents living at high altitude | Wrong outcome |
| Vasquez 2001 | Normal hematological values for healthy persons living at 4000 meters in Bolivia | Wrong outcome |
| Monge 1992 | Pathophysiology and epidemiology of chronic mountain sickness | Not full-text |
| Beall 1999 | Percent of oxygen saturation of arterial hemoglobin among Bolivian Aymara at 3,900-4,000 m | Wrong outcome |
| Zhang 2018 | Periodic breathing persists in healthy high altitude sojourners over time | Wrong outcome |
| Cabada 2010 | Pre-travel Preparation for Cusco, Peru: A Comparison Between European and North American Travelers | Wrong outcome |
| Castro 2013 | Prevalence of sleep-disordered breathing in truck drivers at a mine located at high altitude | Wrong outcome |
| Azad 2021 | Protective role of estrogen against excessive erythrocytosis in Monge disease | Wrong type of study |
| Viruez-Soto 2017 | Pulmonary arterial hypertension and pregnancy at high altitude | Not full-text |
| Allemann 2012 | Pulmonary artery pressure and cardiac function in children and adolescents after rapid ascent to 3,450 m | Not full-text |
|  | Pulmonary edema of high altitude | Not full-text |
| Vincent 1978 | Pulmonary gas exchange, diffusing capacity in natives and newcomers at high altitude | Wrong outcome |
| Antezana 1998 | Pulmonary hypertension in high-altitude chronic hypoxia: Response to nifedipine | Wrong outcome |
| Spliethoff 2013 | Reduced insulin sensitivity as a marker for acute mountain sickness? | Not full-text |
| Rexhaj 2011 | Reproducibility of acute mountain sickness in children and adults: A prospective study | Wrong outcome |
| Santolaya 1989 | Respiratory adaptation in the highest inhabitants and highest Sherpa mountaineers | Not full-text |
| Beall 1992 | Respiratory and hematological adaptations of young and older Aymara men native to 3600M | Wrong outcome |
| Stuber 2008 | Respiratory nitric oxide and pulmonary artery pressure in children of aymara and European ancestry at high altitude | Wrong outcome |
| Bacaloni 2018 | Respiratory parameters at varied altitudes in intermittent mining work | Wrong outcome |
| Loeppky 2005 | Role of hypobaria in fluid balance response to hypoxia | Not full-text |
|  | Ronchopathy and chronic mountain sickness. A case control study | Not full-text |
| Cabada 2009 | Self-reported health problems among travelers visiting Cuzco: A Peruvian Airport survey | Wrong outcome |
| Gonzales 2013 | Serum testosterone levels and excessive erythrocytosis during the process of adaptation to high altitudes | Wrong outcome |
| Martineaud 1969 | The cutaneous circulation during adaptation to high altitude | Wrong outcome |
| Rexhaj 2016 | Sleep-Disordered Breathing and Vascular Function in Patients With Chronic Mountain Sickness and Healthy High-Altitude Dwellers | Wrong outcome |
| Norcliffe 2005 | Cerebrovascular responses to hypoxia and hypocapnia in high-altitude dwellers | Wrong outcome |
| Keyl 2002 | Autonomic cardiovascular function in high-altitude and natives with chronic mountain sickness | Wrong outcome |
| Schwab 2008 | Pulmonary-artery pressure and exhaled nitroxide in bolivian and caucasian high altitude dwellers | Wrong outcome |
| Lazio 2010 | Postexercise peripheral oxygen saturation after completion of the 6-minute walk test predicts successfully reaching the summit of Aconcagua | Wrong outcome |
| Loeppky 2005 | Early fluid retention and severe acute mountain sickness | Wrong outcome |
| Hudson 1999 | The effect of high altitude on platelet counts, thrombopoietin and erythropoietin levels in young Bolivian airmen visiting the Andes | Not full-text |
