# Supplementary material for: Mountain sickness in altitude inhabitants of Latin America: A systematic review and meta-analysis
Source: PLoS One. 2024 Sep 24;19(9):e0305651. doi: 10.1371/journal.pone.0305651 (PMC11421813; doi:10.1371/journal.pone.0305651)
Supplement: S2 Table — (DOCX) [file pone.0305651.s003.docx]

## S2 Table. Search strategy

| **Database** | | | **Search strategy** | |
| --- | --- | --- | --- | --- |
| Pubmed  (767) | #1 | "Altitude Sickness"[Mesh] OR ((Altitude[TIAB] OR Mountain[TIAB] OR highland*[TIAB] OR “high-altitude”[TIAB]) AND (Hypoxia[TIAB] OR Sickness[TIAB] OR diseas*[TIAB])) | |  |
|  | #2 | "Latin America"[Mesh] OR "Latin America*"[TIAB] OR Argentina[Affiliation] OR Bolivia[Affiliation] OR Brasil[Affiliation] OR Chile[Affiliation] OR Colombia[Affiliation] OR “Costa Rica”[Affiliation] OR Cuba[Affiliation] OR Ecuador[Affiliation] OR Guatemala[Affiliation] OR Haiti[Affiliation] OR Honduras[Affiliation] OR Mexico[Affiliation] OR Panama[Affiliation] OR Paraguay[Affiliation] OR “República Dominicana” [Affiliation] OR Uruguay[Affiliation] OR Venezuela[Affiliation] OR Argentina[au] OR Bolivia[au] OR Brasil[au] OR Chile[au] OR Colombia[au] OR “Costa Rica”[au] OR Cuba[au] OR Ecuador[au] OR Guatemala[au] OR Haiti[au] OR Honduras[au] OR Mexico[au] OR Panama[au] OR Paraguay[au] OR Peru[au] OR “República Dominicana”[au] OR Uruguay[au] OR Venezuela[au] | |  |
|  | #3 | #1 AND #2 | |  |
| Scopus  (2275) | #1 | TITLE-ABS-KEY((Altitude OR Mountain OR highland* OR “high-altitude”) AND (Hypoxia OR Sickness OR diseas*)) | |  |
|  | #2 | AFFILCOUNTRY(Argentina OR Bolivia OR Brasil OR Chile OR Colombia OR “Costa Rica” OR Cuba OR Ecuador OR Guatemala OR Haiti OR Honduras OR Mexico OR Panama OR Paraguay OR Peru OR “República Dominicana” OR Uruguay OR Venezuela) | |  |
|  | #3 | #1 AND #2 | |  |
| Web of Science  (350) | #1 | TS=((Altitude OR Mountain OR highland* OR “high-altitude”) AND (Hypoxia OR Sickness OR diseas*)) | |  |
|  | #2 | CU=(Argentina OR Bolivia OR Brasil OR Chile OR Colombia OR “Costa Rica” OR Cuba OR Ecuador OR Guatemala OR Haiti OR Honduras OR Mexico OR Panama OR Paraguay OR Peru OR “República Dominicana” OR Uruguay OR Venezuela) | |  |
|  | #3 | OG=(Argentina OR Bolivia OR Brasil OR Chile OR Colombia OR “Costa Rica” OR Cuba OR Ecuador OR Guatemala OR Haiti OR Honduras OR Mexico OR Panama OR Paraguay OR Peru OR “República Dominicana” OR Uruguay OR Venezuela) | |  |
|  | #4 | #1 AND (#2 OR #3) | |  |
| Embase  (174) | #1 | ('altitude disease'/mj OR 'andes disease':ti,ab,kw OR 'altitude disease':ti,ab,kw OR 'altitude illness':ti,ab,kw OR 'altitude sickness':ti,ab,kw OR 'd`acosta syndrome':ti,ab,kw OR 'high altitude disease':ti,ab,kw OR 'high altitude hypoxia':ti,ab,kw OR 'high altitude illness':ti,ab,kw OR 'high altitude sickness':ti,ab,kw OR 'high altitude syndrome':ti,ab,kw OR 'hypobaropathy':ti,ab,kw OR 'hypoxia, altitude':ti,ab,kw OR 'mountain disease':ti,ab,kw OR 'mountain sickness':ti,ab,kw OR 'soroche':ti,ab,kw) | |  |
|  | #2 | ('argentina':ca OR 'bolivia':ca OR 'brasil':ca OR 'chile':ca OR 'colombia':ca OR 'costa rica':ca OR 'cuba':ca OR 'ecuador':ca OR 'guatemala':ca OR 'haiti':ca OR 'honduras':ca OR 'mexico':ca OR 'panama':ca OR 'paraguay':ca OR 'peru':ca OR 'república dominicana':ca OR 'uruguay':ca OR 'venezuela':ca) | |  |
|  | #3 | #1 AND #2 | |  |
