# Supplementary figures and images for: Mountain sickness in altitude inhabitants of Latin America: A systematic review and meta-analysis
Source: PLoS One. 2024 Sep 24;19(9):e0305651. doi: 10.1371/journal.pone.0305651 (PMC11421813; doi:10.1371/journal.pone.0305651)

## S1 Figure. Risk of bias of included studies.


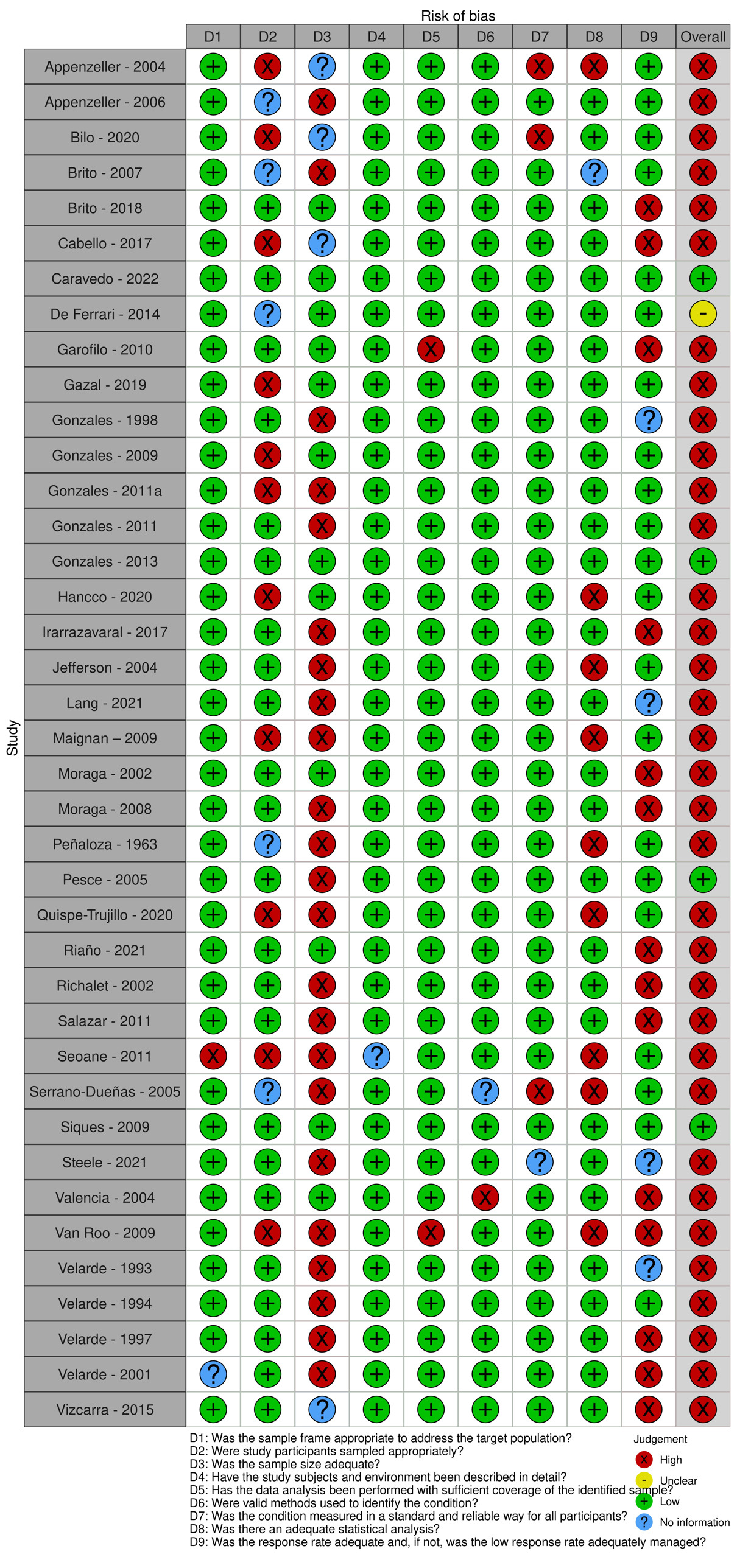

Supplement: S1 Fig — (DOCX) [file pone.0305651.s004.docx]

## S4 Figure. Publication bias on the prevalence of acute (A) and chronic (B) mountain sickness.

AMS


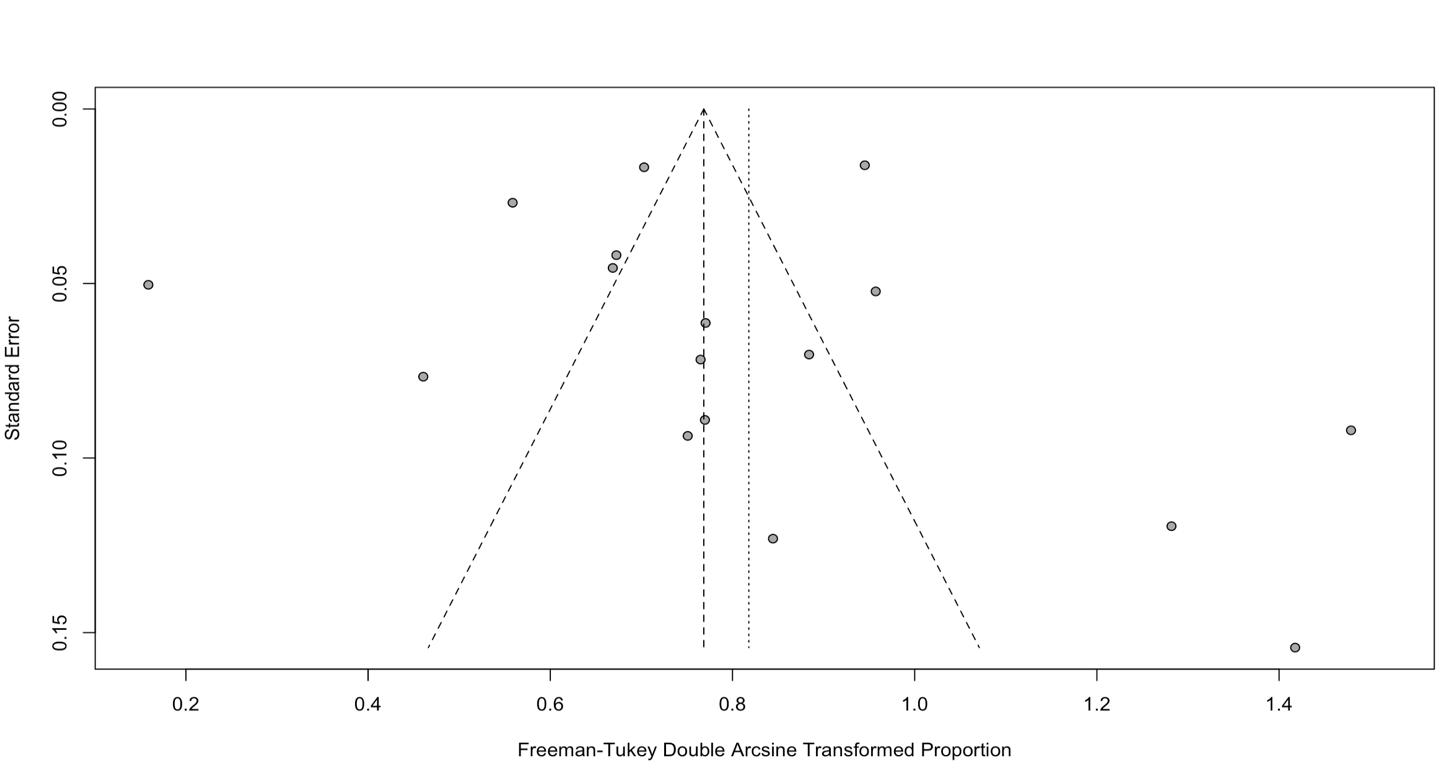


CMS


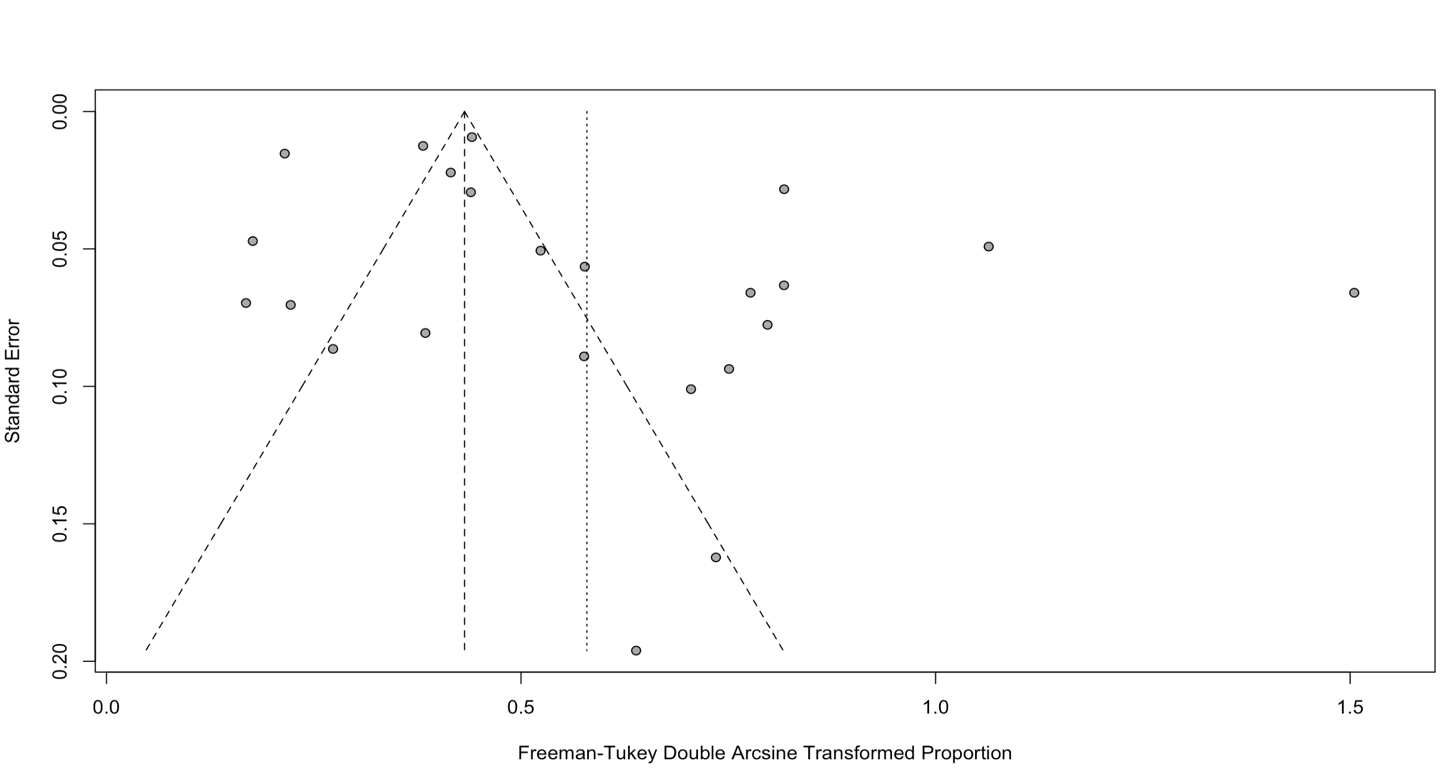

Supplement: S4 Fig — (DOCX) [file pone.0305651.s007.docx]
