# Supplementary material for: Mountain sickness in altitude inhabitants of Latin America: A systematic review and meta-analysis
Source: PLoS One. 2024 Sep 24;19(9):e0305651. doi: 10.1371/journal.pone.0305651 (PMC11421813; doi:10.1371/journal.pone.0305651)
Supplement: S2 Fig — (DOCX) [file pone.0305651.s005.docx]

## S2 Figure. Sensitivity analysis on prevalence of Acute (A) and Chronic (B) Mountain Sickness between risk of bias

**
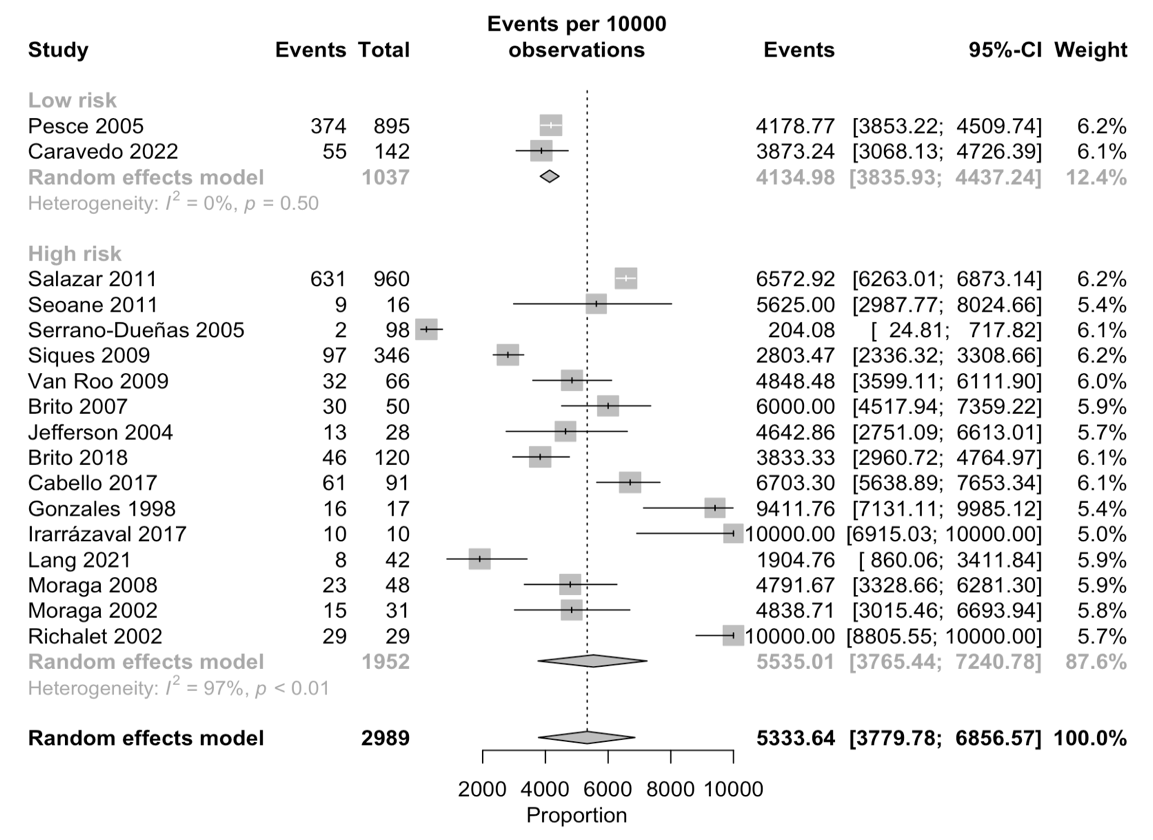
**AMS

**
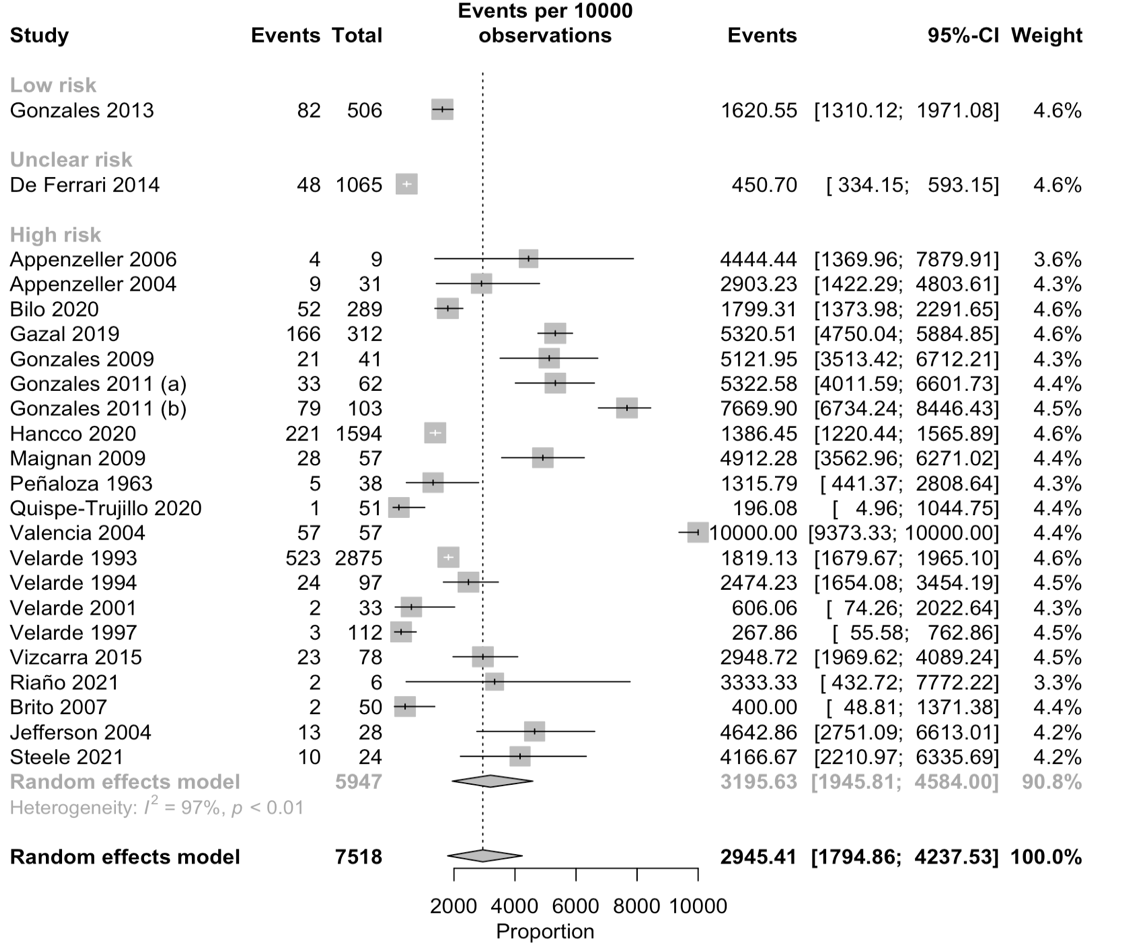
**CMS
