# Supplementary material for: Mountain sickness in altitude inhabitants of Latin America: A systematic review and meta-analysis
Source: PLoS One. 2024 Sep 24;19(9):e0305651. doi: 10.1371/journal.pone.0305651 (PMC11421813; doi:10.1371/journal.pone.0305651)

## S3 Figure. Sensitivity analysis on prevalence of Acute (A) and Chronic (B) Mountain Sickness excluding outliers

AMS
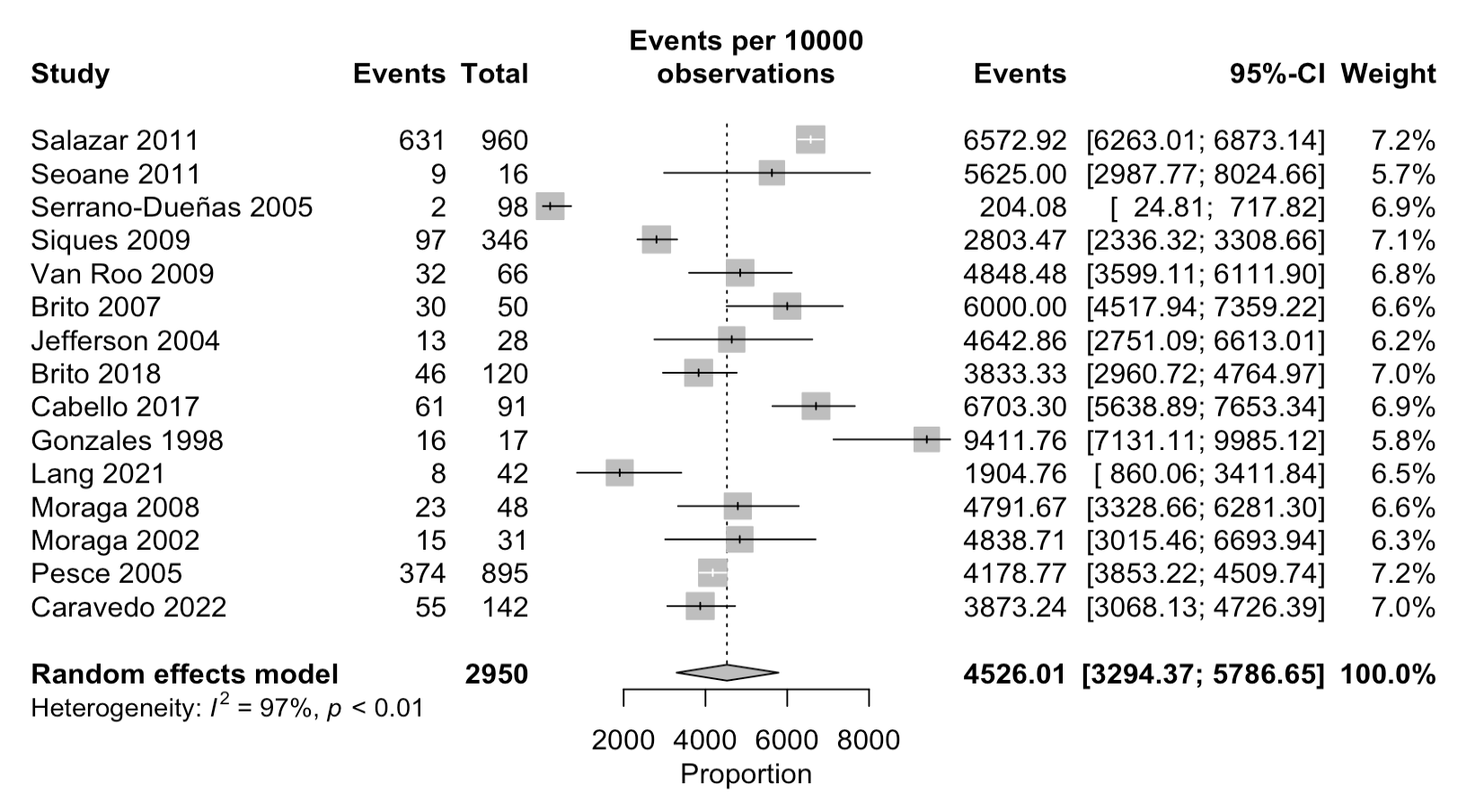


CMS


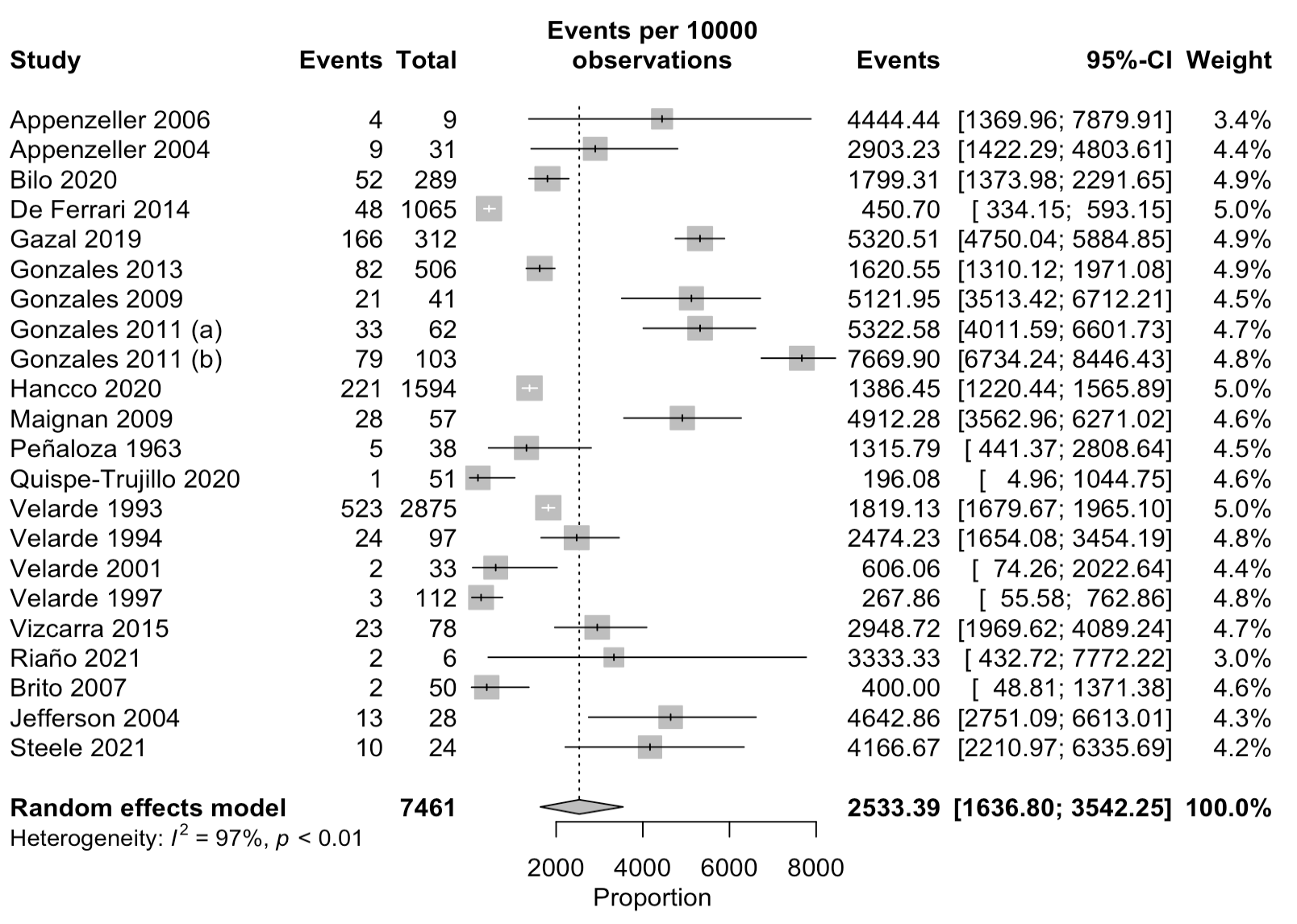

Supplement: S3 Fig — (DOCX) [file pone.0305651.s006.docx]
